# Impact of scale-aware deep convection on the cloud liquid and ice water paths and precipitation using the Model for Prediction Across Scales (MPAS-v5.2)

**DOI:** 10.5194/gmd-13-2851-2020

**Published:** 2020-06-29

**Authors:** Laura D. Fowler, Mary C. Barth, Kiran Alapaty

**Affiliations:** 1 National Center for Atmospheric Research, Boulder, Colorado 80307-3000, USA; 2 Center for Environmental Measurements and Modeling, U.S. Environmental Protection Agency Research Triangle Park, North Carolina 27711, USA

## Abstract

The cloud liquid water path (LWP), ice water path (IWP), and precipitation simulated with uniform- and variable-resolution numerical experiments using the Model for Prediction Across Scales (MPAS) are compared against Clouds and the Earth’s Radiant Energy System (CERES) and Tropical Rainfall Measuring Mission data. Our comparison between monthly-mean model diagnostics and satellite data focuses on the convective activity regions of the tropical Pacific Ocean, extending from the Tropical Eastern Pacific Basin where trade wind boundary layer clouds develop to the Western Pacific Warm Pool characterized by deep convective updrafts capped with extended upper-tropospheric ice clouds. Using the scale-aware Grell–Freitas (GF) and Multiscale Kain–Fritsch (MSKF) convection schemes in conjunction with the Thompson cloud microphysics, uniform-resolution experiments produce large biases between simulated and satellite-retrieved LWP, IWP, and precipitation. Differences in the treatment of shallow convection lead the LWP to be strongly overestimated when using GF, while being in relatively good agreement when using MSKF compared to CERES data. Over areas of deep convection, uniform- and variable-resolution experiments overestimate the IWP with both MSKF and GF, leading to strong biases in the top-of-the-atmosphere longwave and shortwave radiation relative to satellite-retrieved data. Mesh refinement over the Western Pacific Warm Pool does not lead to significant improvement in the LWP, IWP, and precipitation due to increased grid-scale condensation and upward vertical motions. Results underscore the importance of evaluating clouds, their optical properties, and the top-of-the-atmosphere radiation budget in addition to precipitation when performing mesh refinement global simulations.

## 1 Introduction

Comparing simulated against observed global cloud liquid and ice water paths (LWP and IWP) remains challenging because of uncertainties in parameterizing moist processes and cloudiness in global climate and numerical weather prediction (NWP) models and errors in retrieving the LWP and IWP from satellite measurements. Cloud simulations from general circulation models (GCMs) involved in Phase 3 and 5 of the Coupled Model Intercomparison Project (CMIP3; CMIP5; Meehl et al., 2007; Taylor et al., 2012) display a strong disparity in the simulated LWP (Jiang et al., 2012; Li et al., 2018) and IWP (Li et al., 2012), producing annual mean LWP and IWP overestimated by factors of 2 to 10 compared to satellite data. Satellite observations of the LWP and IWP from passive nadir-viewing instruments such as the Moderate-resolution Imaging Spectroradiometer (MODIS; Minnis et al., 2011) and profiling radar such as the 94 GHz instrument on the CloudSat satellite (Stephens et al., 2002) also display major differences among themselves, as discussed in Li et al. (2008) and Waliser et al. (2009). While models and satellite retrievals agree that the LWP and IWP should be defined as the vertically integrated liquid and ice water content, including all non-precipitating and precipitating hydrometeors, this is not always the case in practice, further challenging a clearly posed data–data and model–data comparison. Defining the LWP and IWP varies between models, depending on the complexity of the parameterization of cloud microphysics processes and prognostic versus diagnostic treatment of falling hydrometeors. Defining the measured LWP and IWP varies between satellite products, depending on the sensitivity of the observing systems to detect large precipitating particles. While comparing simulated and observed LWP and IWP may not be as straightforward as comparing the top-of-the-atmosphere (TOA) radiation budget (Dolinar et al., 2015; Stanfield et al., 2015), it offers a different way to directly diagnose biases in simulated total cloud liquid and ice water mass as a first step to help correct deficiencies in parameterizing global-scale moist processes and precipitation.

Before the launch of the CloudSat and Cloud-Aerosol Lidar and Infrared Pathfinder Satellite Observation mission (Stephens et al., 2002), global estimates of the LWP and IWP were retrieved principally from satellite radiance measurements over different spectral intervals (e.g., Alishouse et al., 1990; Greenwald et al., 1993; Minnis et al., 1995; Platnick et al., 2003). In their critical review of most common methods developed to retrieve cloud and precipitation properties from satellite radiances, Stephens and Kummerow (2007) identify two main sources of errors. The first source of errors originates from the mandatory classification between cloudy and cloud-free scenes and between precipitating and non-precipitating cloudy scenes. The second source of errors stems from using forward radiative transfer models that lack details of the vertical distribution of cloudiness and precipitation as well as complexity in specifying the optical properties of liquid water and ice particles. Estimating the LWP and IWP from CloudSat radar reflectivity alone presents its own set of challenges for scenes that include precipitating cloud systems due to the high sensitivity of radar reflectivity to the presence of large particles, for scenes that include mixed-phase and deep convective clouds, and close to the surface due to ground clutter. Li et al. (2018) showed that annual mean maps of MODIS- and CloudSat-based LWP agree relatively well in tropical and subtropical regions if both data sets exclude LWP observations for deep convective/precipitating clouds since MODIS is quite insensitive to precipitation. Stephens and Kummerow (2007) advocate combining satellite-retrieved radar and radiance measurements to help validate simulated cloud properties and precipitation. In addition to considering the impact of precipitating particles, Waliser et al. (2009) demonstrate that a well-posed model–data comparison must include a consistent sampling between model outputs and satellite data to reduce diurnal sampling biases and sensitivity of the sensor and retrieval algorithm to the particle size when computing the simulated LWP and IWP.

Contemporary climate and NWP GCMs (Giorgetta et al., 2018; Molod et al., 2012; Kay et al., 2015; Skamarock et al., 2012) categorize moist processes into three distinct parameterizations, one to simulate turbulent mixing in the planetary boundary layer (PBL) in response to surface forcing and forcing in the free troposphere, one to simulate subgrid-scale shallow and deep convection, and one to include grid-scale cloud microphysics. While coupling between parameterizations varies between GCMs, it is an established practice to let detrained condensates from convective updrafts serve as sources for non-convective grid-scale clouds, as precipitating anvils and cirrus outflow. We suggest that uncertainties in parameterizing moist convection and impact on grid-scale clouds may explain a major part of the differences in the LWP and IWP simulated between the CMIP3 and CMIP5 GCMs. In recent years, efforts have been made to develop unified cloud parameterizations to represent all cloud types and alleviate the need to parameterize complex interactions between stratiform, shallow convective, and deep convective clouds (Guo et al., 2015; Storer et al., 2015; Thayer et al., 2015). Using the global Model for Prediction Across Scales (MPAS; Skamarock et al., 2012), Fowler et al. (2016) discuss the sensitivity of simulated precipitation as spatial resolution increases from hydrostatic to nonhydrostatic scales and suggest to further analyze the associated sensitivity of simulated clouds and TOA radiation. Results show that as subgrid-scale convective motions are increasingly resolved, diagnostic precipitation from the scale-aware Grell–Freitas (GF; Grell and Freitas, 2014) deep convection scheme decreases, while prognostic precipitation from the WSM6 (Hong and Lim, 2006) cloud microphysics scheme increases over the refined area of the variable-resolution mesh. Vertical profiles of the cloud liquid and ice water mixing ratios and cloud fraction highlight the redistribution of cloud condensates and relative humidity with height in the refined area in response to decreased contribution of convective detrainment of cloud liquid water and ice. However, Fowler et al. (2016) do not further address if variations in the vertical profiles of cloud condensates lead to improved LWP, IWP, and cloud optical properties against satellite-derived data.

The objectives of our research are 3-fold. First, we want to assert that our suite of PBL, deep and shallow convection, and cloud microphysics parameterizations tested in MPAS at hydrostatic and nonhydrostatic scales for medium-range spring forecasts over the continental United States (Schwarz, 2019; Wong and Skamarock, 2016) can also be used to produce month-long simulations of tropical convection, narrowing our analysis on the tropical Pacific Ocean. In order to broaden our research and possibly generalize our results, we also implemented the scale-aware Multi-scale Kain–Fritsch (MSKF; Glotfelty et al., 2019; Zheng et al., 2016) parameterization of deep and shallow convection in addition to GF. Second, we want to evaluate the ability of MPAS to simulate the LWP, IWP, cloudiness, and TOA longwave and shortwave radiation against the Clouds and the Earth’s Radiant Energy System (CERES; Wielicki et al., 1996) Single Scanner Foot-Print (SSF; Minnis et al., 2011) data set and precipitation against the Tropical Rainfall Measuring Mission (TRMM) Multisatellite Precipitation Analysis (TMPA; Huffman et al., 2007). Our third goal aims at understanding differences in the LWP, IWP, precipitation, and cloud radiative effects as functions of horizontal resolution with GF and MSKF using the capability of local mesh refinement developed for MPAS.

In Sect. 2, we summarize the characteristics of the GF and MSKF parameterizations of deep and shallow convection. In Sect. 3, we provide a short description of MPAS, including physics parameterizations used with both convective parameterizations, the design of our experiments using the uniform- and variable-resolution meshes, and description of the satellite data sets used to validate our results. In Sect. 4, we analyze our results in terms of precipitation and varying contribution of the convective and grid-scale precipitation to the total precipitation as a function of horizontal resolution. In Sect. 5, we compare the LWP, IWP, and TOA longwave and shortwave radiation against satellite data. In Sect. 6, we discuss some of our findings. Finally, in Sect. 7, we summarize our results and propose areas of future research.

## 2 Description of the convective parameterizations

Mass-flux-based convective parameterizations distinguish themselves through the use of different triggering functions to initiate convection, the details of their entraining-detraining cloud models, and formulation of their closures that control the intensity of convection and computation of the cloud-base mass flux. For convective parameterizations that include deep and shallow convection, criteria that characterize the two kinds of convection strongly vary. Furthermore, how convective parameterizations account for the dependence of convection on the horizontal resolution differs in complexity. In this section, we summarize the chief characteristics of GF and MSKF, including differences in their treatment of deep and shallow convection, and horizontal-scale dependence.

### 2.1 The Grell–Freitas (GF) parameterization

The version of GF used in our numerical experiments is that implemented in version 3.8.1 of the Advanced Research Weather Research Forecast model (Skamarock et al., 2008), as described in Grell and Freitas (2014). Its properties are first discussed in Grell (1993) and later expanded in Grell and Devenyi (2002) to include stochasticism. GF treats deep and shallow convection separately by using different initial entrainment rates (7×10^−5^ and 1×10^−2^ m^−1^ for deep and shallow convection, respectively) to control the depth of convective layers and different closures to calculate the cloud-base mass flux. GF includes an ensemble of closures from well-known convective parameterizations to compute a mean cloud-base mass flux. For deep convection, these four closures are the *AS* closure (Arakawa and Schubert, 1974) that assumes instantaneous equilibrium between the large-scale forcing and subgrid-scale convection; the *W* closure (Brown, 1979; Frank and Cohen, 1987) that relates the cloud-base mass flux to the grid-scale upward vertical velocity; the *MC* closure (Krishnamurti et al., 1983) that calculates the cloud-base mass flux as a function of the vertically integrated vertical moisture advection; and the *KF* closure (Kain and Fritsch, 1993) that reduces the convective available potential energy over a prescribed convective timescale. Qiao and Liang (2015) analyze the separate and combined impacts of the four closures on the simulated summer precipitation over the United States coastal oceans. On the one hand, they find that computing the cloud-base mass flux using the *W* and *MC* closures leads to precipitation patterns and amounts that are in better agreement against TMPA data than those using the *AS* and *KF* closures. On the other hand, they find that the *AS* and *KF* closures yield improved diurnal cycle of precipitation relative to the other two closures. In our numerical experiments, GF gives an equal weight to each closure to calculate the mean cloud-base mass flux for deep convection. As for deep convection, GF includes different closures for shallow convection. In our numerical experiments using GF, we choose the boundary layer quasi-equilibrium (*BLQE*) closure of Raymond (1995) for shallow convection.

Both types of convection transport total water and moist static energy in a conservative manner but neglect to include ice-phase processes in updrafts and downdrafts. In this version of GF, the only feedback between shallow convection and the large-scale environment is lateral and cloud-top detrainment of water vapor and corresponding heating, as liquid water formed in shallow updrafts evaporates immediately. Deep convection returns potential temperature, water vapor, and condensed water tendencies to the environment. Detrained condensed water acts as a source of liquid water (ice) if the large-scale temperature is warmer (colder) than the prescribed 258 K threshold. While GF assumes that shallow convective plumes are not deep enough to produce precipitation, the conversion of liquid water to rain water in deep convective plumes depends on a simple Kesslertype (Kessler, 1969) conversion threshold, and precipitation reaches the surface instantaneously.

**Figure 1.**
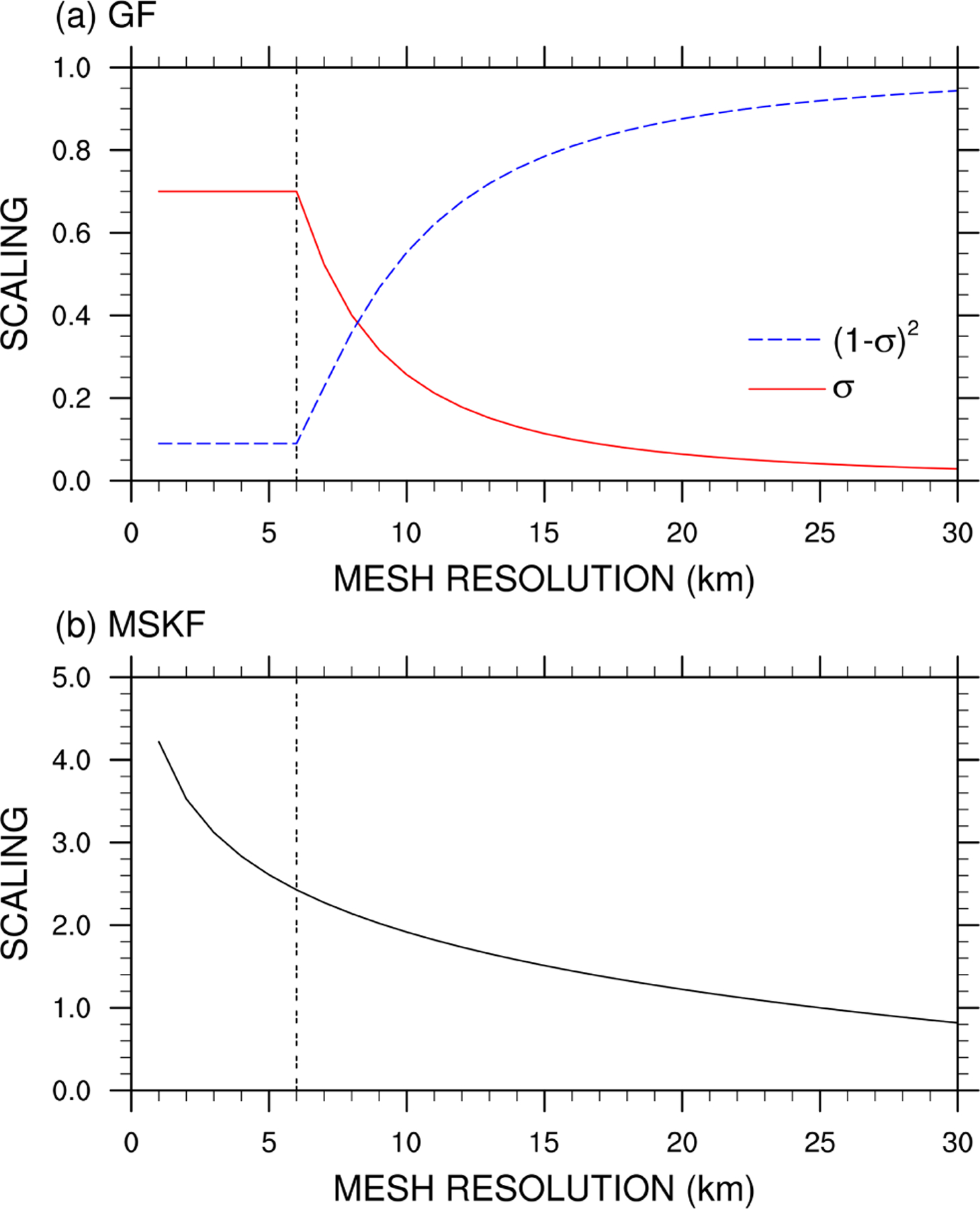
(**a**) Convective updraft fraction as a function of the mesh resolution used to scale the cloud-base mass flux in GF; and (**b**) scaling factor as a function of the mesh resolution used to scale the convective timescale in MSKF.

As discussed in Grell and Freitas (2014), deep convection includes a simplified representation of the unified parameterization of deep convection described in Arakawa and Wu (2013). Arakawa and Wu (2013) demonstrate that mass-flux-based convective parameterizations can be modified to work at all resolutions spanning between hydrostatic and nonhydrostatic scales through the reduction of the convective vertical eddy transport as a quadratic function of the horizontal fraction of the grid box occupied by convective updrafts. In GF, the convective updraft fraction (*σ*) is computed as a simple function of the initial entrainment rate (*ε* = 7×10^−5^ m^−1^) and half-width radius (*R*) of convective updrafts following Simpson and Wiggert (1969), or

(1) $$\begin{array}{l} \sigma =\frac{\pi R^{2}}{A}\\ \text{and}\\ R=\frac{0.2}{\varepsilon }, \end{array}$$

where *A* is the area of the grid box. In Eq. (1), *σ* is not allowed to exceed 0.7, based on the discussion of Grell and Freitas (2014). As discussed in Fowler et al. (2016), when *σ* becomes greater than 0.7, *σ* is set to 0.7 and *ε* is recalculated using Eq. (1), leading to increased entrainment and decreased convective cloud tops as *A* becomes smaller. Another option would be to turn off deep convection when *σ* reaches values close to 1, in which case a better choice for its maximum value may be between 0.9 and 1 (Grell and Freitas, 2014). Figure 1a highlights the rapid decrease in *σ* from 0.7 to 0.3 as spatial resolution decreases from 6 to 9 km. *σ* further decreases from 0.3 to 0.1 for resolutions between 9 and 16 km and from 0.1 to 0.05 for resolutions between 16 and 30km. The (1−*σ*)^2^ quadratic function used to scale the mass flux starts to be significant at resolutions greater than 20 km and decreases rapidly to a minimum value of 0.1 for horizontal grid spacing smaller than 6 km. Using a maximum value for *σ* ensures that over the most refined area of the mesh, parameterized deep convection is not completely turned off since deep convection is not explicitly resolved. Using a variable-resolution mesh varying between 50km over the coarse area of the mesh down to 3km over the refined area of the mesh centered over South America, Fowler et al. (2016) show that the impact of parameterized deep convection weakens and that of grid-scale cloud microphysics strengthens as horizontal grid spacing increases from hydrostatic to nonhydrostatic scales.

### 2.2 The Multi-scale Kain–Fritsch (MSKF) parameterization

MSKF is the scale-aware version of the Kain–Fritsch (KF) convective parameterization, first developed by Kain and Fritsch (1990, 1993) and later updated by Kain (2004) to include, among other improvements, non-precipitating shallow convection. The trigger function is that used in Fritsch and Chappell (1980), originally tested in Kain and Fritsch (1992) and recently in Suhas and Zhang (2014). In MSKF, convection may be triggered if the temperature of a *mixed layer* is greater than that of the environment. The pressure thickness of that mixed layer must be at least 50 hPa thick and is computed as the sum of adjacent layer depths starting at the layer next to the surface. The mixed layer temperature is a pressure-weighted function of the temperatures in those adjacent layers after being lifted to the lifting condensation level (LCL) plus a perturbation temperature linked to the magnitude of the grid-scale vertical motion at the LCL. Once the base of a potential updraft source layer is found, convection remains activated if the vertical velocity of an air parcel lifted using the Lagrangian parcel method remains positive for a minimum cloud depth of 3km, as a test that the convective instability is strong enough for the air parcel to reach the level of free convection (LFC). If not, the procedure is repeated by moving up to the next model layer until a new updraft source layer is found or until the search reaches above the lowest 300 hPa of the atmosphere. Further details on the equations used to compute the perturbation temperature and parcel vertical velocity are found in Kain (2004).

In MSKF, the closure assumption assumes that the convective available potential energy in a cloud layer is removed within a time adjustment period following Bechtold et al. (2001). The convective timescale is defined as the advective timescale in the cloud layer with maximum values of 1h and 40min for deep and shallow convection, respectively. In contrast to GF, the thermodynamics inside the cloud model includes the ice phase. The condensed water formed in each cloudy layer is partitioned between liquid water and ice, assuming a linear transition of the cloud temperature between 268 and 248K. A fraction of the condensed water converts to rain, following Ogura and Cho (1973), and reaches the ground instantaneously. As discussed in Kain (2004), when an updraft source layer is identified, the classification of a convective cloud layer as deep or shallow depends on the cloud depth. Shallow convection is activated when all the criteria for deep convection are met, but the depth of the updraft is shallower than the minimum cloud depth (3km). This definition implies that shallow and deep convection are not allowed to coexist. In the case of shallow convection, precipitation formed in updrafts is detrained to the environment as rain or snow, providing an additional moisture source to the large-scale environment. As in GF, MSKF provides tendencies of temperature, water vapor, and cloud liquid water/ice to the environment and tendencies of rain and snow from shallow convection.

MSKF contains many improvements over KF, as summarized in the Supplement of Glotfelty et al. (2019). These improvements include subgrid-scale cloud feedbacks to radiation from both shallow and deep convection leading to more realistic surface downward radiation, as described in Alapaty et al. (2012), and the scale dependence of fundamental parameters so that MSKF can be used at spatial resolutions varying between hydrostatic and nonhydrostatic scales. As detailed in Glotfelty et al. (2019) and Zheng et al. (2016), MSKF uses a scale-dependent formulation (*β*) to the adjustment timescale (*τ*) for deep and shallow convection based on Bechtold et al. (2008), or

(2) $$\begin{array}{l} \tau =\frac{H}{W_{\text{cl}}}\beta \\ \text{and}\\ \beta =1+\ln\left(\frac{25}{\Delta x}\right), \end{array}$$

where *H* and *W*_cl_ are the depth of the convective cloud and cloud-averaged vertical velocity, and Δ*x* is the grid spacing. Figure 1b highlights the dependence of the *β* scaling parameter as a function of horizontal resolution. As many MSKF parameters are optimized for a resolution around 25km (Kain, 2004), *β* is equal to 1 at 25km, ramping up to values greater than 2.4 for resolutions higher than 6 km. Because the adjustment timescale is proportional to *β* (Zheng et al., 2016), it increases as horizontal resolution increases, leading to scale-aware stabilization of the atmosphere by MSKF. In addition, MSKF includes a new scale-aware formulation of the minimum entrainment rate using the LCL as a function of the scale-dependent *Tokioka* parameter (Tokioka et al., 1988), a scale-dependent conversion rate for liquid water and ice condensates to precipitation, an increased grid-scale velocity expressed in terms of the subgrid-scale updraft mass flux, and elimination of double counting of precipitation in cloudy layers. The separate and combined impacts of the development of MSKF on high-resolution weather forecasts and regional climate simulations are discussed in Herwehe et al. (2014), Mahoney (2016), He and Alapaty (2018), Zheng et al. (2016), and Glotfelty et al. (2019).

## 3 Methodology

### 3.1 Numerical experiments

We discuss differences in our MPAS results between GF and MSKF configurations on precipitation, cloud properties, and TOA radiation using 30 d long numerical experiments in MPAS (Skamarock et al., 2012). MPAS is a global nonhydrostatic atmospheric model developed for NWP and climate studies. The horizontal discretization uses an unstructured spherical centroidal Voronoi tessellation with a C-grid staggering, as described in Ju et al. (2011), while the vertical discretization is the height-based hybrid terrain-following coordinate of Klemp (2011). The dynamical solver integrates the prognostic equations (cast in flux form) for the horizontal momentum, vertical velocity, potential temperature, dry air density, and scalars using the split-explicit technique of Klemp et al. (2007). The temporal discretization uses a third-order Runge-Kutta scheme and the explicit time-splitting technique described in Wicker and Skamarock (2002). We use the monotonic option of the scalar transport scheme of Skamarock and Gassmann (2011) for horizontal and vertical advection of all moist scalars on the unstructured Voronoi mesh. Finally, horizontal filtering of the state variables is based on Smagorinsky (1963), as described in Skamarock et al. (2012). For variable-resolution meshes, the eddy viscosity coefficient is scaled as a function of the inverse mesh density so that horizontal diffusion is increased in the coarse area relative to the refined area of the mesh.

In MPAS, the computational flow includes three distinct steps. The first step calls the physics parameterizations that update the surface energy budget and calculate the tendencies of potential temperature, moist species, and zonal and meridional wind due to longwave and shortwave radiation, subgrid-scale convection, condensation and mixing in the PBL and free troposphere, and gravity wave drag due to orography. The physics parameterizations use the same input surface boundary conditions and soundings to compute their respective tendencies. Besides GF and MSKF, these parameterizations are

- the Noah land surface parameterization described by Chen and Dudhia (2001),
- the longwave and shortwave Rapid Radiative Transfer Model for GCMs (RRTMG) described by Mlawer et al. (1997) and Iacono et al. (2000),
- the semiempirical parameterization of the cloud fraction of grid-scale clouds from Xu and Randall (1996) and convective clouds from Xu and Krueger (1991) for use in the longwave and shortwave RRTMG schemes (following Xu and Randall, 1996, the fractional amount of grid-scale clouds is a function of the relative humidity and grid-averaged condensate mixing ratio of cloud liquid water, ice, and snow; in MSKF, the fractional amount of shallow and deep convective clouds depends on the convective mass flux),
- the Mellor–Yamada–Nakanishi–Niino (MYNN) planetary boundary layer (PBL) and surface layer scheme described by Nakanishi and Niino (2009) with many updates described in Olson et al. (2019), and
- the gravity wave drag parameterization of Hong et al. (2008).

The second step calls the dynamical solver, which updates the state variables with their respective diabatic tendencies in conjunction to applying horizontal and vertical advection. Finally, the third step calls the grid-scale cloud microphysics parameterization so that at the end of the model time step, supersaturation has been entirely removed or the relative humidity does not exceed 100%. Unlike the physics parameterizations listed in step one, the grid-scale cloud microphysics scheme updates the potential temperature and moist species for the next time step instead of providing individual tendencies. The bulk cloud microphysics parameterization of Thompson et al. (THOM; 2004, 2008) is used in all our numerical experiments. THOM includes prognostic equations for temperature, mass mixing ratio of water vapor, cloud liquid water, rain, cloud ice, snow, and graupel and number concentration of cloud ice and rain. We set the number concentration of cloud droplets to 300×10^6^ m^−3^ over land and 100×10^6^ m^−3^ over oceans. In RRTMG, we diagnose the radiative effective radii of cloud liquid water, cloud ice, and snow as functions of the THOM cloud particle assumptions to add coupling between the cloud microphysics and cloud optical properties, as discussed in Thompson et al. (2016).

To compare the two convective parameterizations against satellite-derived data at hydrostatic scales, we use a uniform-resolution mesh for which the mean distance between cell centers is 30km, corresponding to 655 362 cells. The vertical scale includes 55 layers with monotonically increasing thicknesses varying from 50m next to the surface to 700m below 10km to 1000m below the model top over ocean cells. The model top is set at 30km. The dynamics and physics time steps are both set to 150s, and the horizontal diffusion length scale is set to 30km. Longwave and shortwave radiation is called every 15 min, and THOM is cycled twice so that the cloud microphysics time step is less than 90s to ensure computational stability (Greg Thompson, personal communication, 2017). With each convection scheme, we have performed a 1-month long experiment preceded by a 2d spinup to simulate Northern Hemisphere early-winter, initializing our experiments with ERA-Interim (Dee et al., 2011) reanalyses for 00:00 UTC on 29 November 2015. ERA-Interim sea-surface temperatures and sea ice fractions are used to update ocean cells daily. We refer to uniform-resolution experiments run with GF and MSKF as GFu and MSKFu, respectively.

### 3.2 Sensitivity experiments

**Figure 2.**
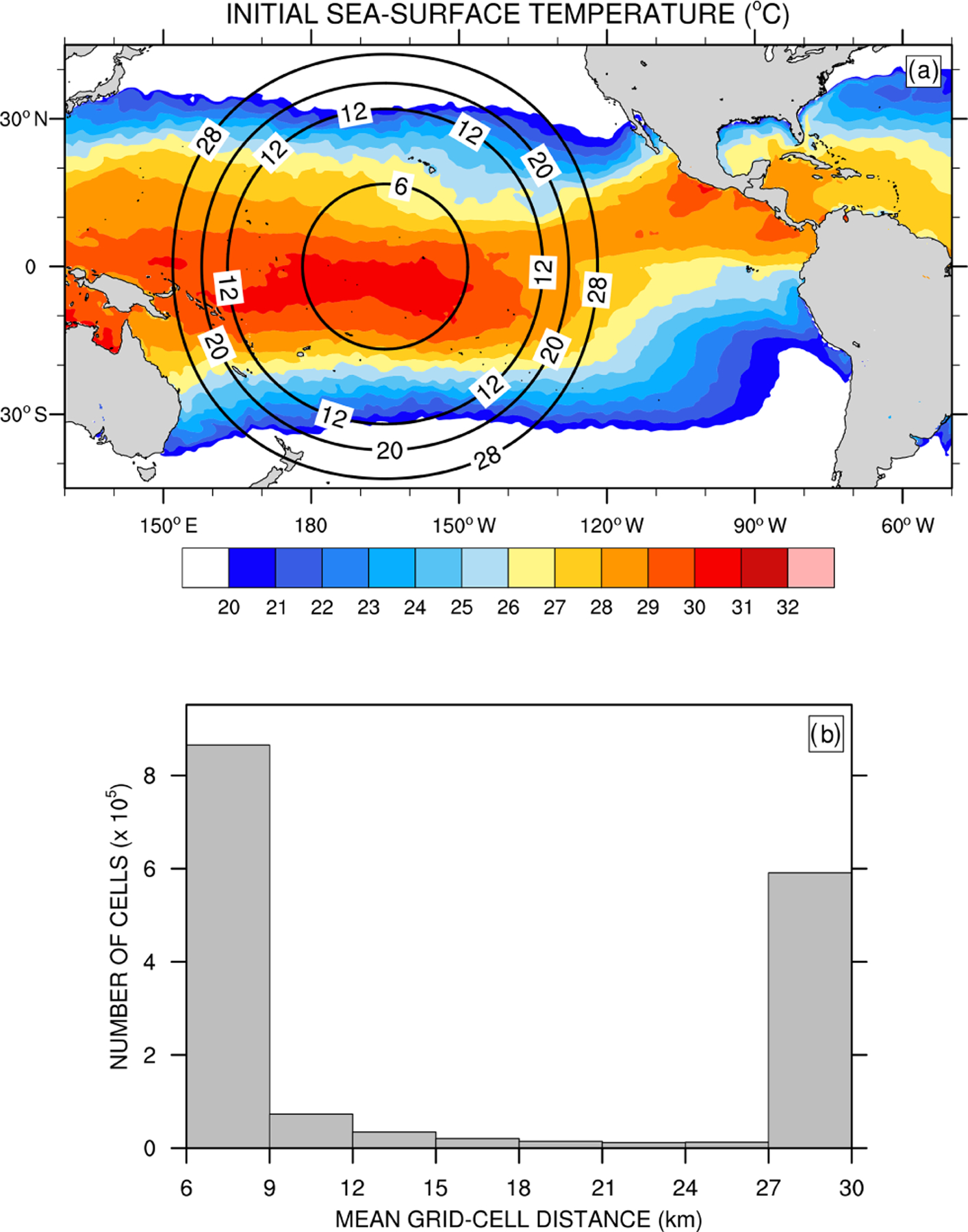
(**a**) Initial sea-surface temperature and refined variable-resolution mesh depicted using isolines of the mean distance between grid-cell centers (km) over the tropical Pacific Ocean; and (**b**) histogram of the number of cells as a function of the mean distance between grid-cell centers.

Using a variable-resolution mesh spanning between 50 and 3km in MPAS, Fowler et al. (2016) demonstrate that subgrid-scale convection parameterized with GF weakens and grid-scale cloud microphysics parameterized with WSM6 (Hong and Lim, 2006) strengthens as resolution increases from the coarse to the most refined area of the mesh. Over the most refined area, grid-scale precipitation contributes a major part to total precipitation, and vertical profiles of subgrid-scale deep convective heating and drying resemble those obtained with a precipitating shallow convection scheme. Fowler et al. (2016) suggest investigating the effect of variable resolution on cloud macrophysical properties and TOA radiation, as grid-scale cloud microphysics parameterizations provide a more physically based description of condensation and precipitation over the refined area of the mesh, compared to simpler entraining-detraining cloud models used in parameterized convection schemes. With the aim to quantify changes in cloud properties and radiation across scales using GF and MSKF, we repeat the early-winter experiments but with a variable-resolution mesh that spans between 30 and 6 km and includes 1622018 cells. As shown in Fig. 2a, we center the refined area of the mesh over the Pacific warm pool defined as the area of the western Pacific Ocean where sea-surface temperatures (SSTs) exceed 28.5 °C, or between 170° E and 140° W. East of 140° W, the north–south width of warmest SSTs across the transition zone between the refined and coarse mesh narrows to delineate the location of the Inter-Tropical Convergence Zone (ITCZ) in the Tropical Eastern Pacific. West of 170° E, the end of mesh refinement borders the eastern tip of Papua New Guinea. Along the Equator, the transition zone between nonhydrostatic and hydrostatic scales spans 20° in the meridional direction on either side of the most refined area of the mesh.

Figure 2b displays a histogram of the mean distance between grid-cell centers. Differences between the initialization of the variable- and uniform-resolution experiments include a reduced time step from 150 to 30 s and a reduced minimum horizontal diffusion length scale from 30 to 6 km. Also, THOM is called only once per physics time step. We refer to our variable-resolution experiments run with GF and MSKF as GFv and MSKFv, respectively. Differences between GFu, GFv, MSKFu, and MSKFv are listed in Table 1. We acknowledge that running single 30 d long experiments is a nontraditional way to assess the performance of convective parameterizations in an NWP framework but is needed to provide increased satellite sampling when comparing simulated clouds and precipitation against observations. Judt (2020) computed the predictability of the atmosphere using global convection-permitting simulations with the same version of MPAS as in this study but with a global uniform mesh with a 4 km cell spacing. Results show that the predictability of the tropics (*>*20d) is longer than that of the extratropics and polar regions (∼2 weeks) when deep convection is mostly resolved. Using the Center for Ocean-Land-Atmosphere Studies GCM with a triangular T63 truncation and the relaxed Arakawa–Schubert parameterization of deep convection (Moorthi and Suarez, 1992), Strauss and Paolino (2008) demonstrate greater predictability in the tropics than in the extratropics at hydrostatic scales. As our comparison between experiments and satellite data focuses on the tropical Pacific Ocean, we are confident that biases arising during the first 2 weeks persist at longer timescales and remain clearly depicted in their monthly means. In order to further assess the robustness of our results, we also compare the 30 d versus 10 d mean LWP, IWP, and precipitation to ensure that biases discussed in Sects. 4 and 5 are qualitatively similar as those observed at shorter timescales (not shown for brevity).

### 3.3 Satellite data sets

We compare the cloud liquid water path (LWP) and ice water path (IWP), cloud area fraction (CF), and the top-of-the-atmosphere longwave upward (TOALW) and shortwave net (TOASW) radiation simulated in our numerical experiments against the Edition 4 Single Scanner Footprint (SSF) products from the Clouds and the Earth’s Radiant Energy System (CERES; Wielicki et al., 1996). Minnis et al. (2011) describe in great detail the retrieval of simultaneous and collocated radiation fluxes and cloud properties from the CERES radiometers and the Moderate-resolution Imaging Spectroradiometer (MODIS) using consistent algorithms and calibration across satellite platforms and shared auxiliary input (temperature and humidity profiles). SSF data are available in two different formats. The first data file format contains 1h of radiation fluxes and cloud properties at the instantaneous CERES 20 km footprint level from the sun-synchronous afternoon (morning) equatorial crossing time Aqua (Terra) satellites. As illustrated in Minnis et al. (2011; their Fig. 15), the CF in each SSF is given in terms of a clear fraction, a fraction for an upper and lower cloud layer separately, and a fraction for an upper layer over a lower layer, although the overlap CF is not available and set to zero in the Edition 4 release version that we are using. The LWP, IWP, and all other cloud fields are provided for the lower and upper layers, separately.

**Figure 3.**
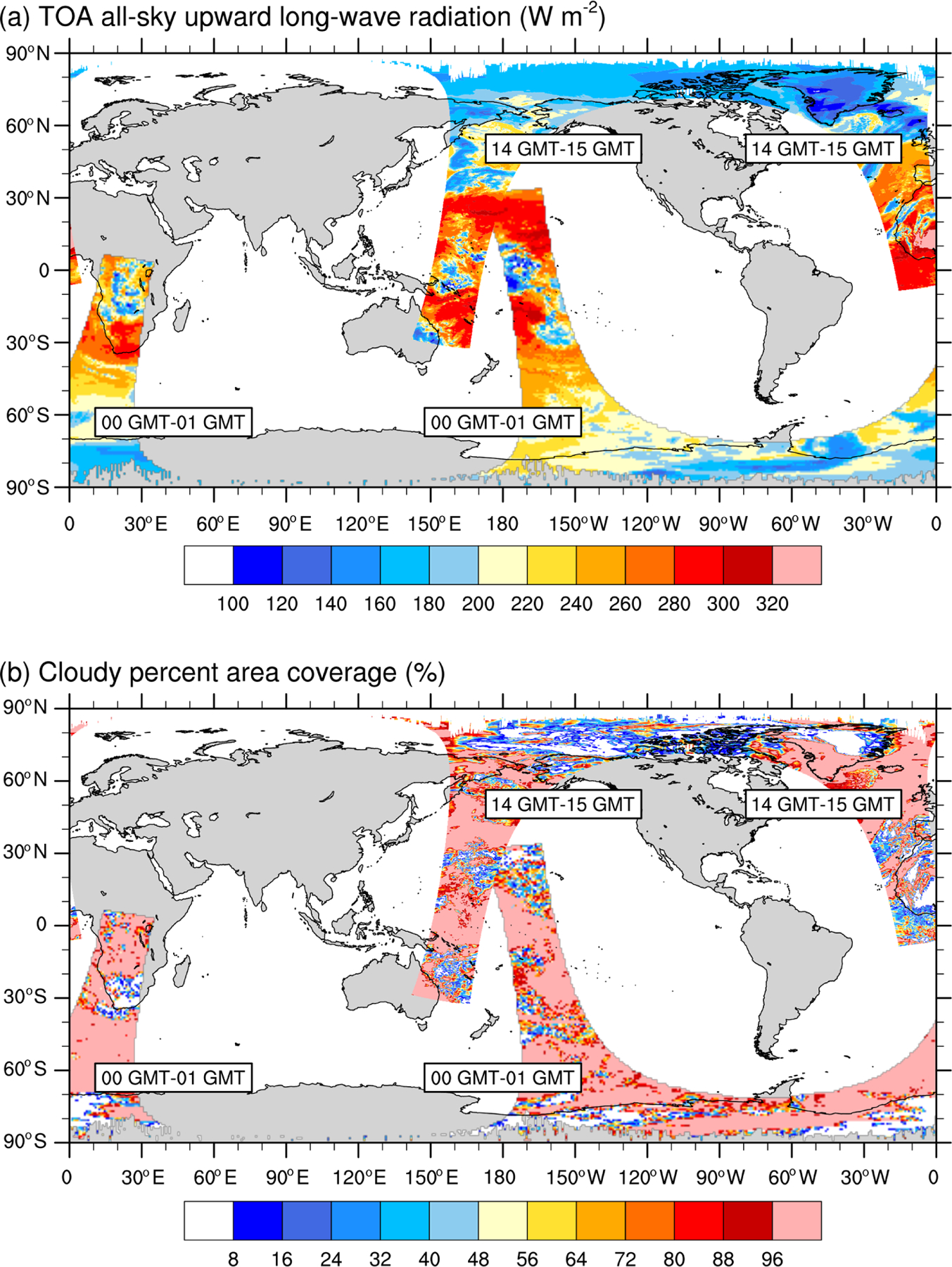
Orbital paths of the Aqua satellite between 00:00 and 01:00 and 14:00 and 15:00GMT after binning the SSF data onto a 0.2°×0.2° rectangular grid for (a) the TOA all-sky upward longwave radiation and (b) the cloudy percent area coverage for 1 December 2015.

Figure 3 illustrates two orbits of the Aqua satellite, one between 00:00 and 01:00GMT and one between 14:00 and 15:00GMT, showing the TOALW (a) and CF (b), after gridding the hourly orbital data to a 0.2°×0.2° latitude–longitude grid. Gridded radiation fluxes and cloud data are means over all SSF data contained inside each rectangular grid, after applying a linear interpolation to reduce the number of missing values. Missing values, highlighted in gray in all figures, depict rectangular grids that did not contain radiation and cloud data in any of the SSF inside the 0.2°×0.2° grid. As seen in Fig. 3, our gridding of the orbital data removes most of the missing data along each orbit, providing a clear depiction of the relationship between the TOALW and CF for cloudy and cloud-free grid cells. Areas of high (low) TOALW coincide with areas of small (large) cloudy areas, but it is also interesting to note that some areas in each orbit are characterized as overcast in conjunction with areas that are not as spatially uniform in TOALW as in CF.

The second data file format (SSF1deg) includes daily and monthly averages of the original SSF orbital data but interpolated on a 1°×1° latitude–longitude grid. The difficulty in using hourly higher-resolution orbital data instead of monthly-mean lower-resolution 1°×1° latitude–longitude gridded product is that the former are available in two distinct *dynamic* layers, while the latter is provided at fixed pressure levels and for the atmospheric column. The lower and upper layers are referred to as *dynamic* layers because the cloud-top (base) pressure of each layer varies between SSFs along each orbit. The advantage of using orbital hourly data is that they can be gridded and interpolated to a spatial resolution close to that of our uniform- and variable-resolution numerical experiments prior to computing monthly-mean radiation and cloud fields. We choose the 0.2°×0.2° latitude–longitude gridded hourly data derived from the first data file format through the entire paper.

**Figure 4.**
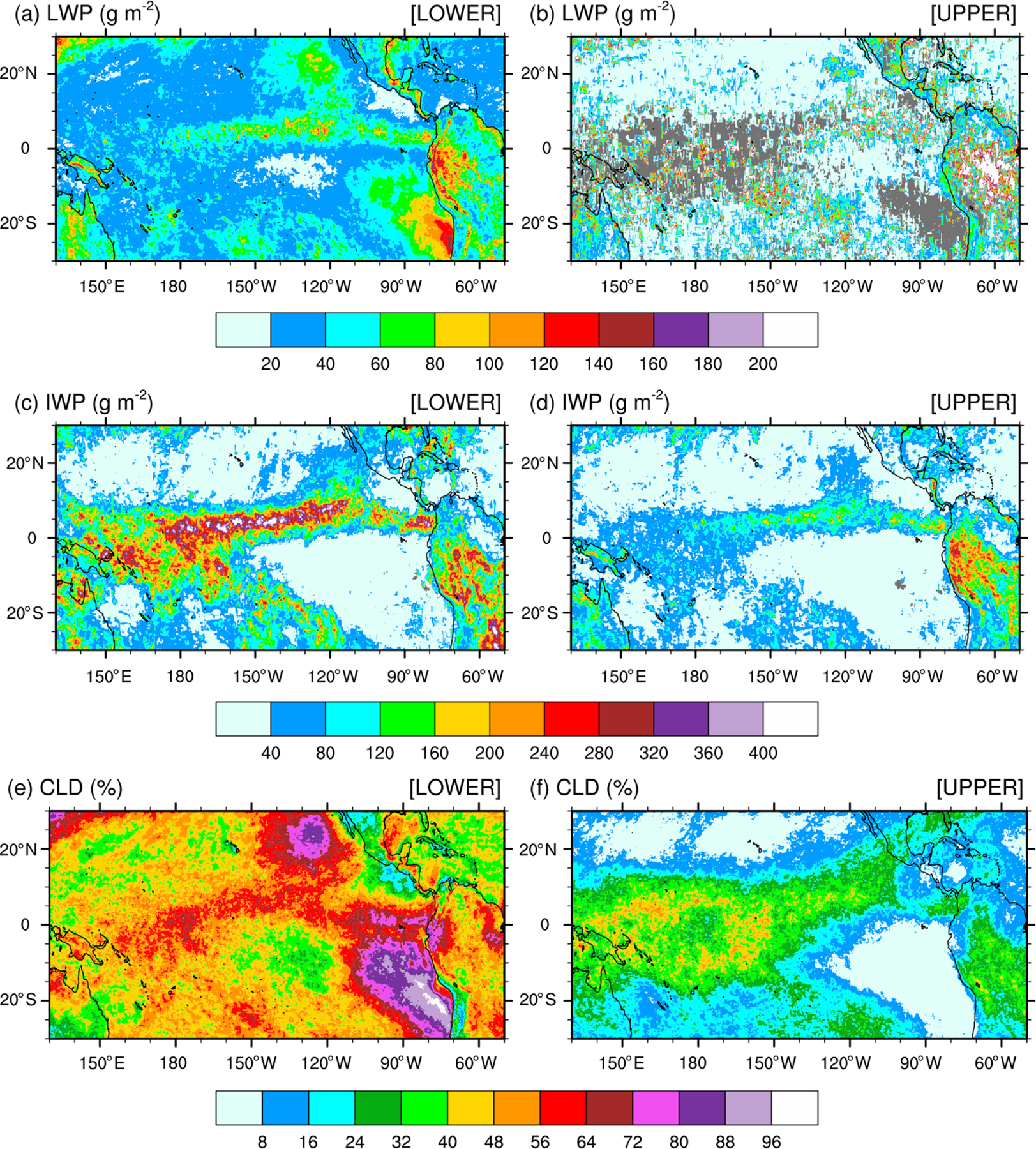
(**a, b**) Monthly-mean cloud liquid water path (LWP), (**c, d**) cloud ice water path (IWP), and (**e, f**) cloud fraction (CLD) over the tropical Pacific Ocean for December 2015 from the Aqua satellite. Panels (**a**), (**c**), and (**e**) are for the lower cloud layer; panels (**b**), (**d**), and (**f**) are for the upper cloud layer.

In order to best compare the simulated against satellite-derived LWP and IWP, we need to understand the partitioning of the SSF LWP and IWP between the two cloud layers. In brief, a lower and an upper cloud layer can be detected simultaneously if they lie adjacent to each other inside an SSF. In that case, the cloud properties for each layer are reported separately. In the case when an opaque upper cloud layer is detected to be above a lower cloud layer, it is impossible to identify the two layers separately. Then, only one cloud layer is reported and always classified as the lower cloud layer, regardless of its cloud-base (top) pressure (Norman Loeb, personal communication, 2019). Further details on the cloud classification, including determination of the cloud phase, are found in Geier et al. (2003) and Minnis et al. (2011). Figure 4 shows the monthly-mean LWP, IWP, and CF for the lower (a, c, e) and upper (b, d, f) layer measured by Aqua for December 2015 over the tropical Pacific Ocean. Figure S1 is as Fig. 4 but for the Terra satellite (see the Supplement). LWP and IWP are *in-cloud* values meaning that they have not been weighted by CF. The lower cloud layer includes stratiform clouds that form over colder sea-surface temperatures along the coast of Peru and off the Baja California Peninsula. Over these areas of CF greater than 72% for the lower cloud layer, CF for the upper cloud layer is less than 8%, highlighting that a single layer of low-level clouds fills a major fraction of the SSF. Increased values of CF are seen in conjunction with increased (decreased) values for the LWP (IWP) in the lower cloud layer indicative of warm-phase clouds, as well seen as off the coast of Peru. High values for the CF and IWP juxtaposed with lower values for the LWP in the lower cloud layer depict clearly deep convection over the eastern Pacific Ocean, ITCZ, and warm pool region. Over areas of deep convection, upper cloud layers are often detected in conjunction with lower cloud layers within the same SSF but are defined by decreased values for the CF and IWP. For the LWP, the coexistence of a lower and upper cloud layer is quite infrequent, as seen by the number of missing grid points in Fig. 4b (Fig. S1b). Where detected, the LWP in the upper layer exceeds that in the lower layer, indicative of warm-phase mature thicker cumulus clouds coexisting with developing thinner cumulus clouds in the lower layer. Finally, outside of the typical stratus cloud regions and either sides of the ITCZ and warm pool region, SSF data reveal extended regions of warm-phase thinner clouds characteristic of widespread shallow convection over tropical oceans.

**Figure 5.**
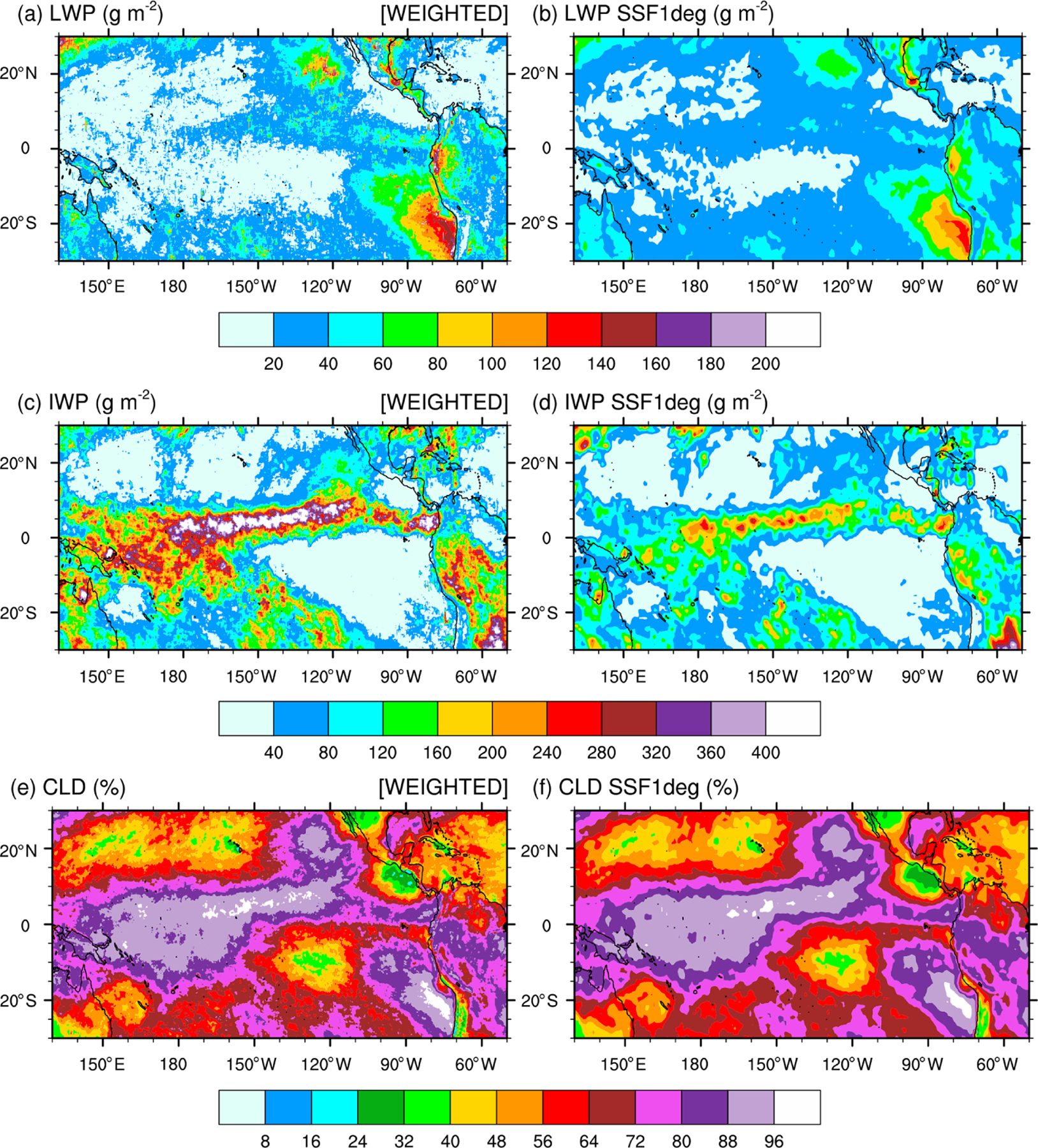
(**a, b**) Monthly-mean cloudy area-weighted cloud liquid water path (LWP), (**c, d**) cloud area-weighted cloud ice water path (IWP), and (**e, f**) cloud fraction (CLD) over the tropical Pacific Ocean for December 2015. Panels (**a**), (**c**), and (**e**) are SSF data; panels (**b**), (**d**), and (**f**) are SSF1deg climatological data.

Calculating the satellite-retrieved LWP and IWP in an atmospheric column for validation of those from our numerical simulations is a two-step process. Because simulated LWPs and IWPs are *grid-cell mean* values and not *local* values, we first multiply the SSF LWP and IWP by CF to get their mean values in the lower and upper cloud layers separately, prior to gridding the hourly orbital data. Second, because the lower and upper layers are defined as adjacent to each other and never overlap in an SSF, we simply add the grid-cell mean LWP and IWP in the lower layer to that in the upper layer to compute the total LWP and IWP. Our processing method is simpler than the processing steps taken by the CERES Science Team to spatially grid and temporally average SSF hourly orbital data to SSF1deg gridded monthly-mean data. Figure 5 compares the monthly-mean 0.2°×0.2° latitude–longitude CF-weighted LWP and IWP and CF (a, c, e) against the SSF1deg products (b, d, f) for December 2015 over the tropical Pacific Ocean. Figure 5a and b show that our method reproduces successfully the geographical patterns and magnitude of the LWP over the tropical Pacific when compared against the SSF1deg data for both months. In contrast, because our method does not weigh the IWP as a function of height, it systematically overestimates the SSF IWP when compared against the SSF1deg data, as seen over the ITCZ and South Pacific Convergence Zone (SPCZ) in both months.

Using ice water content data from the ascending (daytime) and descending (nighttime) portion of CloudSat orbits, Waliser et al. (2009; Fig. 7) estimate that day–night fluctuations in the ice water content at 215 hPa account for as much as 13% (20%) of the annual mean ice water content over the warm pool (Tropical Eastern Pacific), in response to the diurnal cycle of deep convection over the tropical oceans. Therefore, when computing the monthly-mean CF, LWP, IWP, TOALW, and TOASW produced with GFu, GFv, MSKFu, and MSKFv, we first sample the hourly model diagnostics in accordance with the Aqua and Terra satellite orbits in order to reduce biases from different diurnal sampling between our experiments and SSF data. Because the MODIS-based retrieval of the LWP and IWP is insensitive to precipitation, and the rain, snow, and graupel mixing ratios are prognostic variables in THOM and fall through the atmosphere at finite velocities, we infer that the LWP and IWP must include all precipitating and non-precipitating condensates.

In addition to CERES SSF data, we use the monthly-mean precipitation rates from the TRMM Multisatellite Precipitation Analysis (TMPA Version 7; Huffman et al., 2007) to compare simulated versus observed precipitation rates, and monthly-mean ERA-Interim reanalyses (Dee et al., 2011) to compare simulated versus observed precipitable water in the lower troposphere.

## 4 Simulated versus satellite-retrieved precipitation

### 4.1 Incidence of subgrid-scale shallow and deep convection

**Figure 6.**
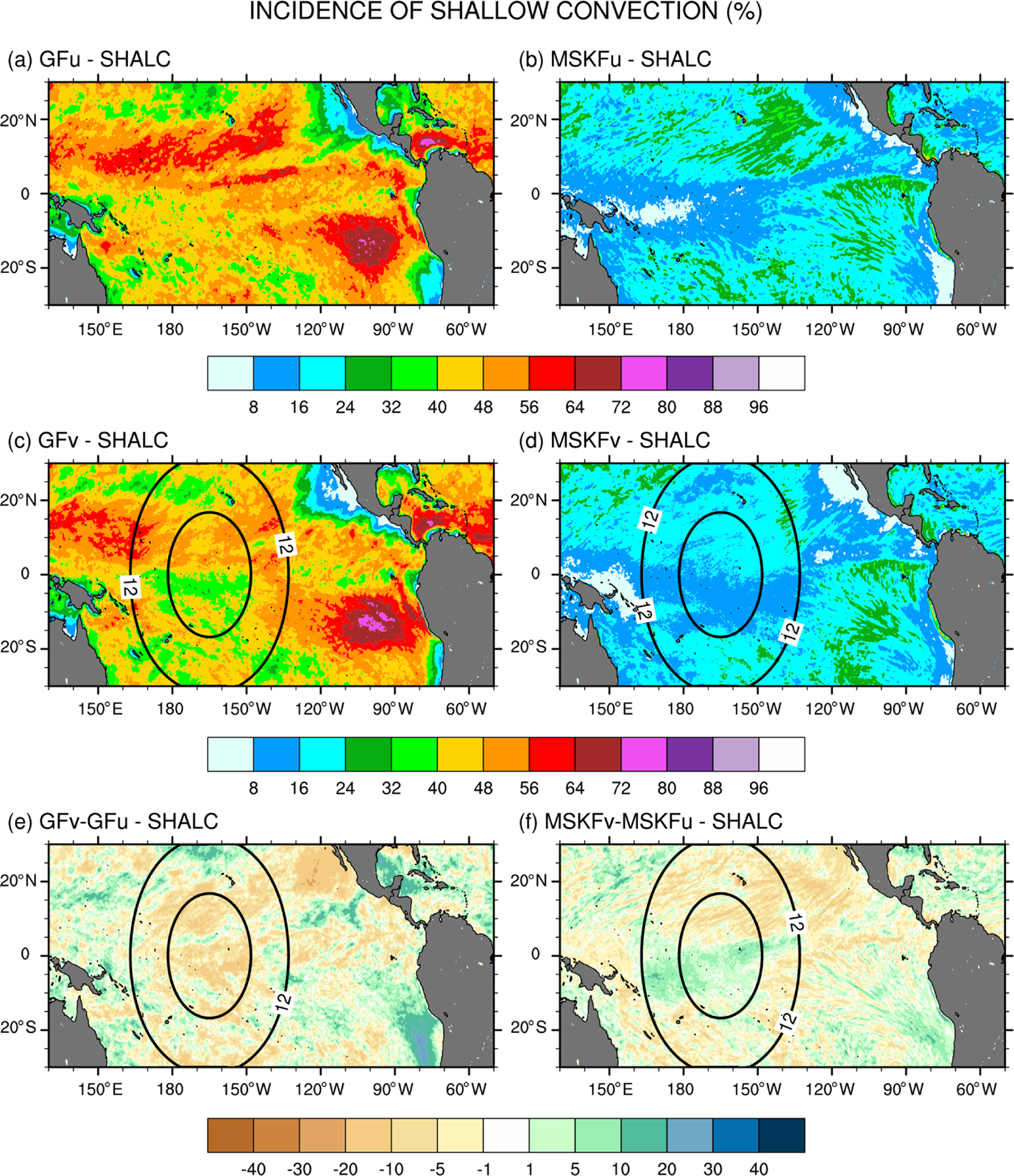
Monthly-mean incidence of shallow convection (SHALC) over the tropical Pacific Ocean simulated in (**a, b**) GFu and MSKFu and (**c, d**) GFv and MSKFv and difference in the incidence of shallow convection between (**e**) GFv and GFu and (**f**) MSKFv and MSKFu for December 2015.

**Figure 7.**
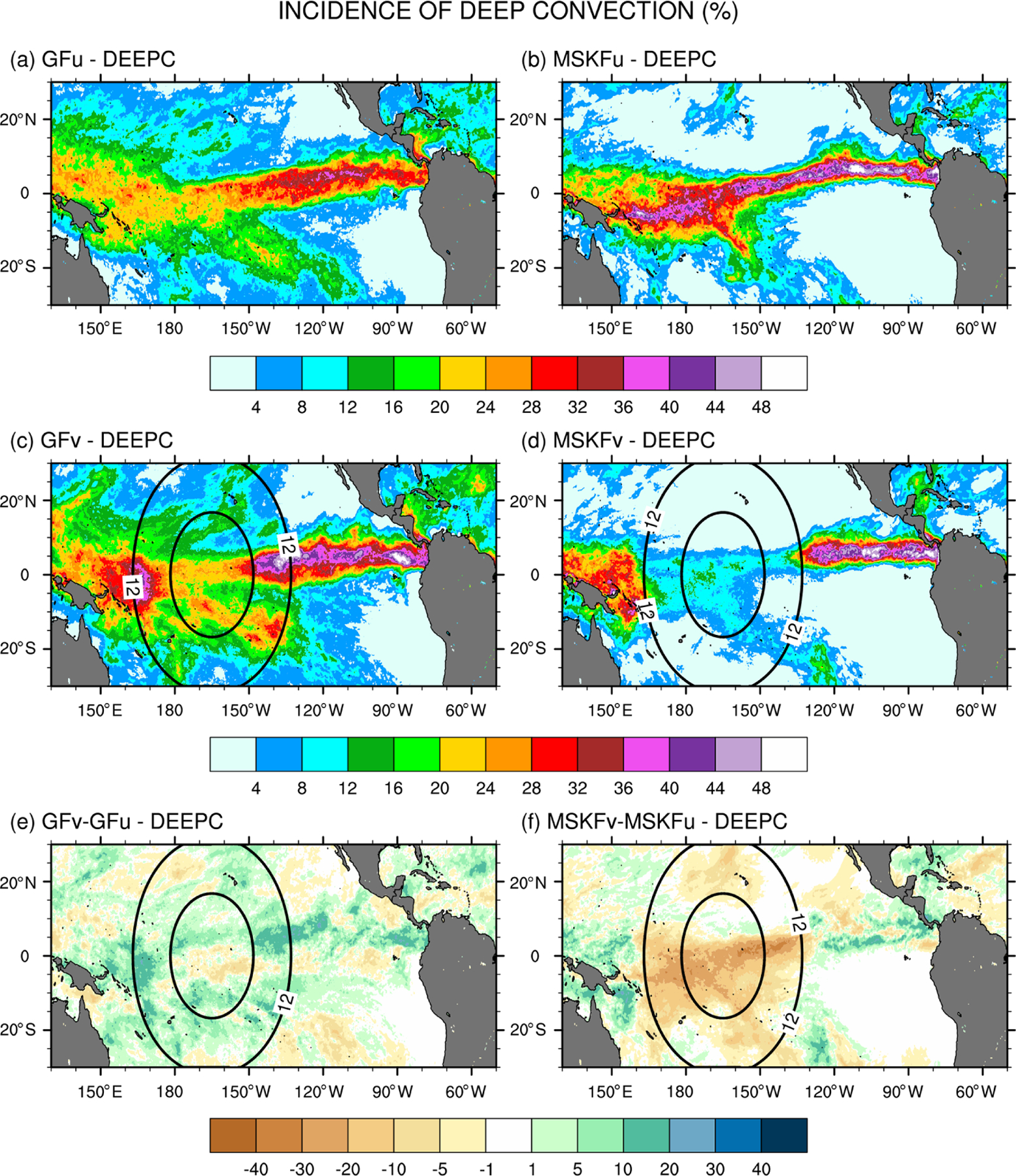
As Fig. 6 but for the monthly-mean incidence of deep convection (DEEPC).

Differences in the treatment of interactions between shallow and deep convection in GF and MSKF, as described in Sect. 2, are bound to modify the partitioning between shallow and deep convection as spatial resolution increases over the refined area of the mesh. A useful diagnostic to analyze the response of shallow and deep convection to local mesh refinement is the incidence of convection. Because shallow convection in both GF and MSKF is non-precipitating, we set the incidence of shallow convection to 100% when cloud tops of shallow convective updrafts are detected and 0% otherwise. We set the incidence of deep convection to 100% when convective precipitation occurs and 0% otherwise. Figures 6 and 7 highlight the impact of the horizontal scale dependence of convection on the monthly-mean incidence of subgrid-scale shallow and deep convection in our uniform- and variable-resolution experiments for December 2015.

Figure 6 shows that simulated shallow convection occurs over the entire tropical Pacific and that its incidence is about twice as large in GFu and GFv as in MSKFu and MSKFv. In GFu and GFv, incidence in excess of 48% covers most of the tropical Pacific, including the ITCZ and warm pool where GF allows shallow and deep convection to occur simultaneously. GFu and GFv exhibit highest incidence of shallow convection off the coast of Peru where persistent low-level stratiform clouds are formed. In contrast, the incidence of shallow convection in MSKFu and MSKFv never exceeds 32% over the entire domain and is less than 16% over the ITCZ and warm pool where shallow and deep convection are not allowed to coexist in MSKF. Figure 6e and f highlight differences in the incidence of shallow convection between GFv and GFu and MSKFv and MSKFu. Despite the fact that GF does not include a spatial scale dependence in its formulation of shallow convection, GFv produces reduced shallow convection relative to GFu over most of the tropical Pacific, except most notably immediately off the coast of Peru. In contrast to GFv, MSKFv yields increased incidence of shallow convection over most of the warm pool region. In MSKF, the height of deep convective clouds decreases as horizontal resolution increases. As the classification between deep and shallow convection is a function of cloud depth, convective clouds originally defined as deep are reclassified as shallow, leading to increased incidence of shallow convection in the refined area of the mesh.

Figure 7a–d show that, in contrast to shallow convection, the incidence of deep convection has the same order of magnitude in GFu and MSKFu and GFv and MSKFv. The top panels (a, b) reveal that the incidence of deep convection is higher in MSKFu than in GFu over the ITCZ and warm pool. In MSKFu, a sharp transition between areas of high and low incidence of deep convection causes areas outside of the ITCZ and warm pool to be mostly void of deep convection, as seen between 10 and 30° N. In GFu, the incidence of deep convection is decreased over the warm pool relative to the ITCZ west of 160° W. Outside of the ITCZ and warm pool, GFu and GFv lead to higher incidence of deep convection than MSKFu and MSKFv because, in contrast to MSKF, GF allows deep and shallow convection to coexist in the same grid cell. Middle panels (c, d) highlight decreased incidence of subgrid-scale deep convection inside the refined area of the mesh over the warm pool in both GFv and MSKFv, as we expect clouds to be resolved on the higher-resolution grid, in conjunction with increased incidence east and west of the refined area. The decreased incidence in the refined area is more pronounced between MSKFu and MSKFv than between GFu and GFv, whereas the upscaling impact of spatial refinement outside the refined area is greater in GFv than in MSKFv. The scale-aware formulation in GF does not produce the same contrast between the refined and coarse mesh in GFv and GFu as that in MSKFv and MSKFu. Figure 7f reveals a reduced incidence in excess of 25% between MSKFu and MSKFv starting at resolutions higher than 12 km flanked by increased incidence of deep convection east and west of the refined area. In contrast, Fig. 7e displays a longitudinal band of decreased incidence of deep convection between 90° W and the dateline, bordered by increased deep convection north of the Equator and south of 10° S. Table 2 lists the area-averaged incidence of deep and shallow convection for an area inside the refined mesh (REFINED: 0.1–5.1° N; 150–180° W) and an area over the Tropical Eastern Pacific (EAST: 3.1–8.1° N; 90–120° W), as later shown in Fig. 9a. The REFINED and EAST areas display little variation in the incidence of shallow convection between GFu (MSKFu) and GFv (MSKFv), but the incidence of shallow convection in GFu and GFv is much higher than in MSKFu and MSKFv. The incidence of subgrid-scale deep convection is higher in the EAST area compared to the REFINED area in all four experiments. Over the REFINED area, the incidence of subgrid-scale deep convection remains about the same between GFu and GFv but strongly decreases between MSKFu and MSKFv.

As described in Sect. 2, MSKF differentiates shallow from deep convection as a function of the convective cloud depth. As spatial resolution increases, the scale-aware formulation leads to a reduction in the intensity of convection and depth of convective clouds, mostly deep convection, over the refined area as seen in Fig. 7f. As the depth of convective clouds originally classified as precipitating deep convective clouds becomes shallower, MSKF reclassifies those same clouds as non-precipitating shallow clouds, leading to near-equal compensation between the decreased and increased incidence of deep and shallow convection over the warm pool. In contrast to MSKF, GF causes precipitating deep convection to become precipitating shallow convection at increased spatial resolution. As this process occurs in the deep convection scheme, and both cloud types precipitate, variations in the incidence of deep convection between GFu and GFv are small. Further analysis of the response of shallow convection between GFu and GFv over the refined area is beyond the objectives of this research.

### 4.2 Precipitation rates

**Figure 8.**
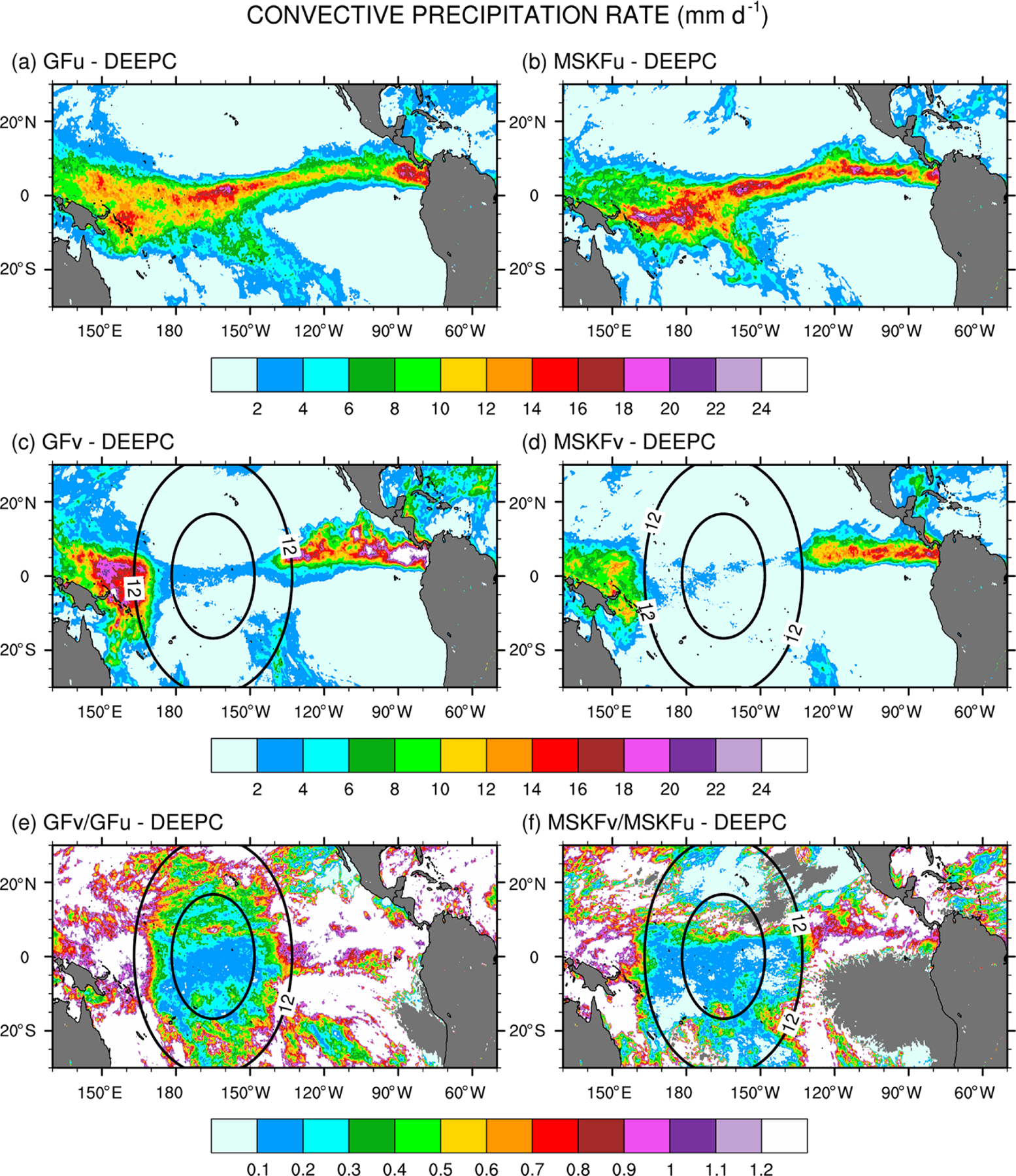
Monthly-mean convective (DEEPC) precipitation rate over the tropical Pacific Ocean simulated in (**a, b**) GFu and MSKFu and (**c, d**) GFv and MSKFv and ratio between the monthly-mean convective precipitation rate in (**e, f**) GFv (MSKFv) and GFu (MSKFu) for December 2015.

Figure 8 shows the monthly-mean convective precipitation rate simulated in GFu and MSKFu (a, b) and GFv and MSKFv (c, d). Figure 8e and f display the ratio between the convective precipitation rate simulated in GFv (MSKFv) and GFu (MSKFu) to contrast the impact of the scale-aware formulation in GF and MSKF. The top panels (a, b) highlight similar geographical patterns of convective precipitation in GFu and MSKFu. Between 80 and 160° W, increased convective precipitation is located along the ITCZ, in conjunction with increased incidence of deep convection, as seen in Fig. 7a–b. West of 160° W, GFu leads to decreased but more widespread convective precipitation relative to MSKFu over the warm pool, in conjunction with decreased but more widespread incidence of convection. In GF, this result infers that while deep convection is not triggered as often over the warm pool as along the ITCZ, the amount of convective precipitation produced in one time step is higher over the warm pool than along the ITCZ, so that monthly-mean convective precipitation rates remain about the same in both regions. Figure 8c and d, in agreement with Fig. 7c and d, display a strong decrease in convective precipitation in both GFv and MSKFv over the refined area of the mesh. In MSKFv, the strong reduction in convective precipitation occurs not only over the most refined area of the mesh but also where horizontal grid spacing increases from 6 to 12 km. In GFv, convective precipitation increases sharply as soon as grid spacing is greater than 12 km and exceeds that simulated in GFu over the coarse area of the mesh. In GFv, the monthly-mean convective precipitation rate is higher than that in MSKFv over the most refined area of the mesh but starts to increase more rapidly between 6 and 12 km than in MSKFv. Differences in increasing convective precipitation across the transition zone between the refined and coarse areas reflect different impacts of the scale-aware formulation in GF and MSKF. Figure 8e and f show that the ratio in convective precipitation between GFv and GFu has the same order of magnitude as that between MSKFv and MSKFu over the refined area of the mesh. While it remains as small in the transition zone as in the refined mesh with MSKF, this ratio increases to values greater than 1 between 6 and 12 km with GF, indicating increased convective precipitation on each side of the refined area in GFv relative to GFu, as also seen in Fig. 8c. Maps of monthly-mean grid-scale precipitation rates show similar geographical patterns between GFu and MSKFu. Over the refined area, increased grid-scale precipitation compensates decreased convective precipitation in both GFv and MSKFv. Over the coarse area, grid-scale precipitation decreases along the ITCZ and warm pool in GFv, while remaining nearly the same in MSKFv (not shown for brevity).

**Figure 9.**
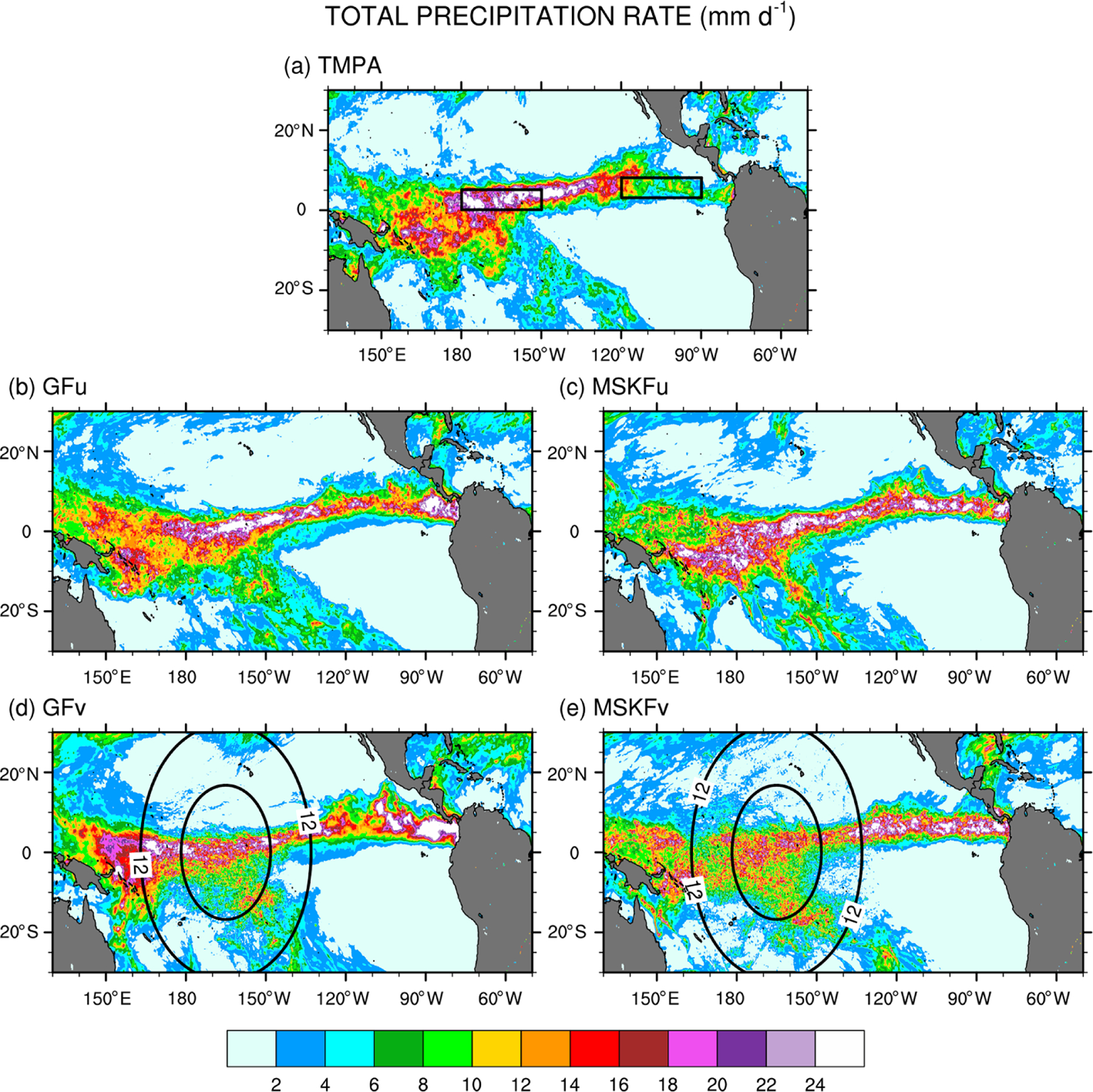
Monthly-mean total precipitation rate over the tropical Pacific Ocean from (**a**) TMPA data and simulated with (**b, c**) GFu and MSKFu and (**d, e**) GFv and MSKFv for December 2015.

**Figure 10.**
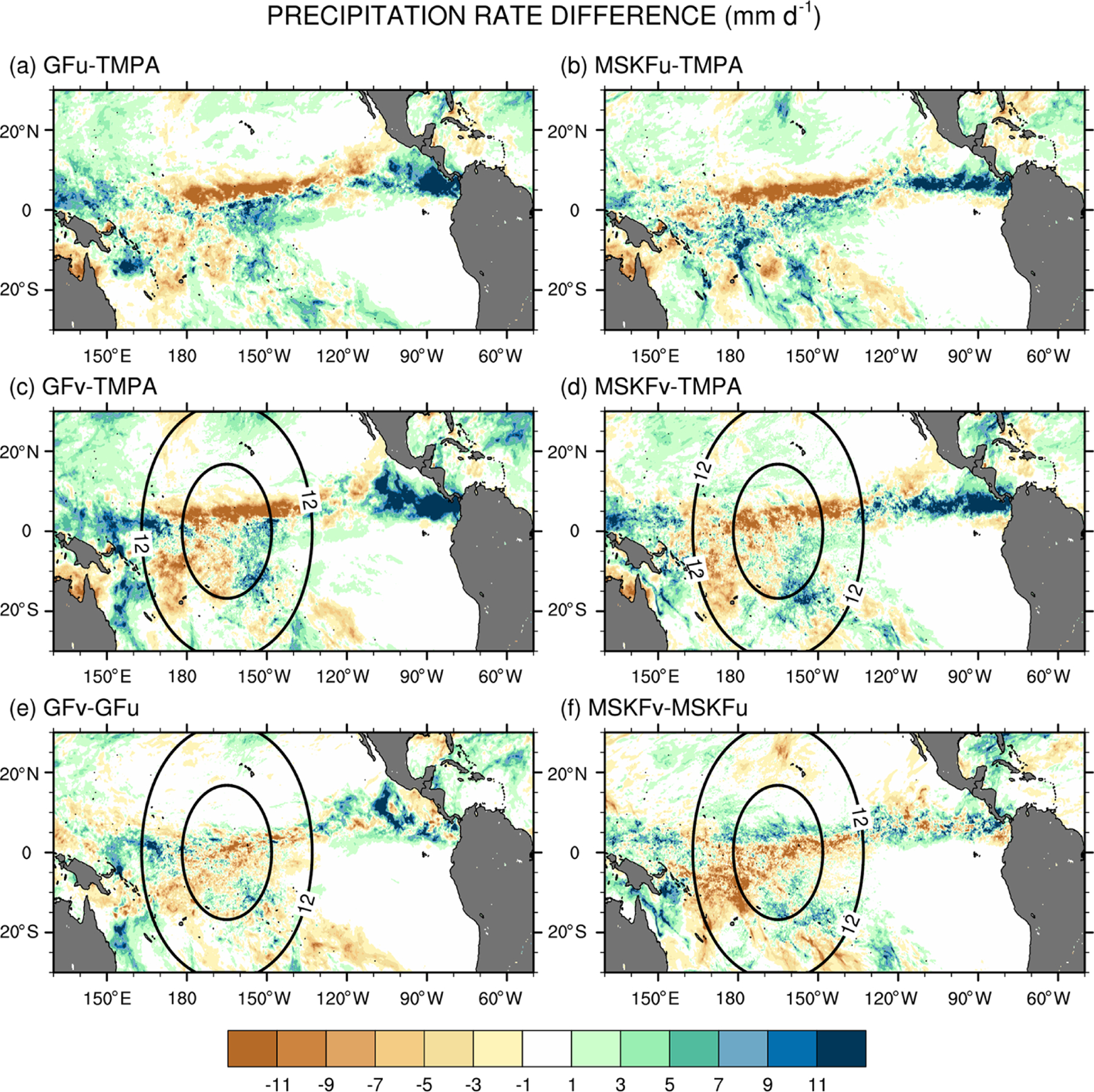
Monthly-mean precipitation rate difference over the tropical Pacific Ocean between (**a, b**) GFu (MSKFu) and TMPA data, (**c, d**) GFv (MSKFv) and TMPA data, and (**e, f**) GFv (MSKFv) and GFu (MSKFu) for December 2015.

The simulated total precipitation rate can be compared to observed TMPA precipitation using Figs. 9 and 10, which show the precipitation rates and differences between simulated and observed precipitation rates, respectively. Areas of maximum satellite-retrieved precipitation are found over the ITCZ between 130° W and the dateline (Fig. 9a). Observed precipitation decreases over the warm pool west of the dateline and decreases strongly over the Tropical Eastern Pacific (between 80 and 120° W) and the SPCZ. The four simulations overestimate precipitation in the Tropical Eastern Pacific between 80 and 120° W (Fig. 9.b–e) with biases in excess of 11mmd^−1^ (Fig. 10a–d). The four simulations also overestimate precipitation between 130 and 160° E, or west of the refined area, with biases about as large as those seen east of the refined area, except for MSKFu. The uniform-grid results (Fig. 9b–c) display the highest precipitation rates over the area of warmest SSTs where we expect deepest convection to occur and are in reasonable agreement with TMPA data. However, GFu and MSKFu locate the ITCZ south of its observed location (Figs. 10a, b), producing a positive bias straddling the Equator and a negative bias north of the Equator. The scale-aware dependence of deep convection in GF leads to decreased total precipitation in GFv compared to GFu over the entire refined area (Fig. 10e). In contrast, Fig. 10f shows that while the scale-aware dependence in MSKF leads to decreased precipitation in MSKFv over a major fraction of the refined area, it also leads to an improved location of the simulated ITCZ, as evidenced by increased precipitation north of the Equator.

Table 3 summarizes the area-mean monthly-mean convective, grid-scale, and total simulated and observed TMPA precipitation rates over the REFINED and EAST areas. Over the two areas, the simulated total precipitation is about the same for all four experiments but is underestimated (overestimated) relative to TMPA data over the REFINED (EAST) areas, respectively. Over the REFINED area, total precipitation decreases by 2.1mmd^−1^ between GFu and GFv and by 2.3mmd^−1^ between MSKFu and MSKFv, highlighting a near-equal compensation between decreased deep convective and increased grid-scale precipitation over the most refined area of the mesh. Over the EAST area, total precipitation increases by 2.7mmd^−1^ between GFu and GFv resulting from a 5.3 (2.6)mmd^−1^ increase (decrease) in convective (grid-scale) precipitation. In contrast, total precipitation increases by 1.2mmd^−1^ between MSKFu and MSKFv resulting from a 0.5 (0.6)mmd^−1^ increase in convective (grid-scale) precipitation. The large (small) increase in convective precipitation in GFv (MSKFv) over the coarse areas east (and west) of the refined area highlights distinct upscaling effect of the refined area on the coarse area of the mesh between GFv and MSKFv.

In summary, the scale dependence of convection in GF and MSKF produces the same partitioning between convective and grid-scale precipitation inside the refined area or decreased convective and compensating increased grid-scale precipitation as horizontal resolution increases. The upscaling impact on convective and grid-scale precipitation varies between GF and MSKF. As seen in Fig. 8 and Table 3, convective precipitation increases strongly over the warm pool and eastern Pacific starting across the transition zones east and west of the refined area in GFv. In contrast, while the parameterization of the scale dependence of deep convection in MSKF produces a stronger decrease in convective precipitation in MSKFv than in GFv, it produces a smoother transition in convective precipitation and decreased upscaling effect as spatial resolution reaches 30km.

## 5 Simulated relative humidity and simulated versus satellite-retrieved LWP and IWP

### 5.1 Relative humidity

**Figure 11.**
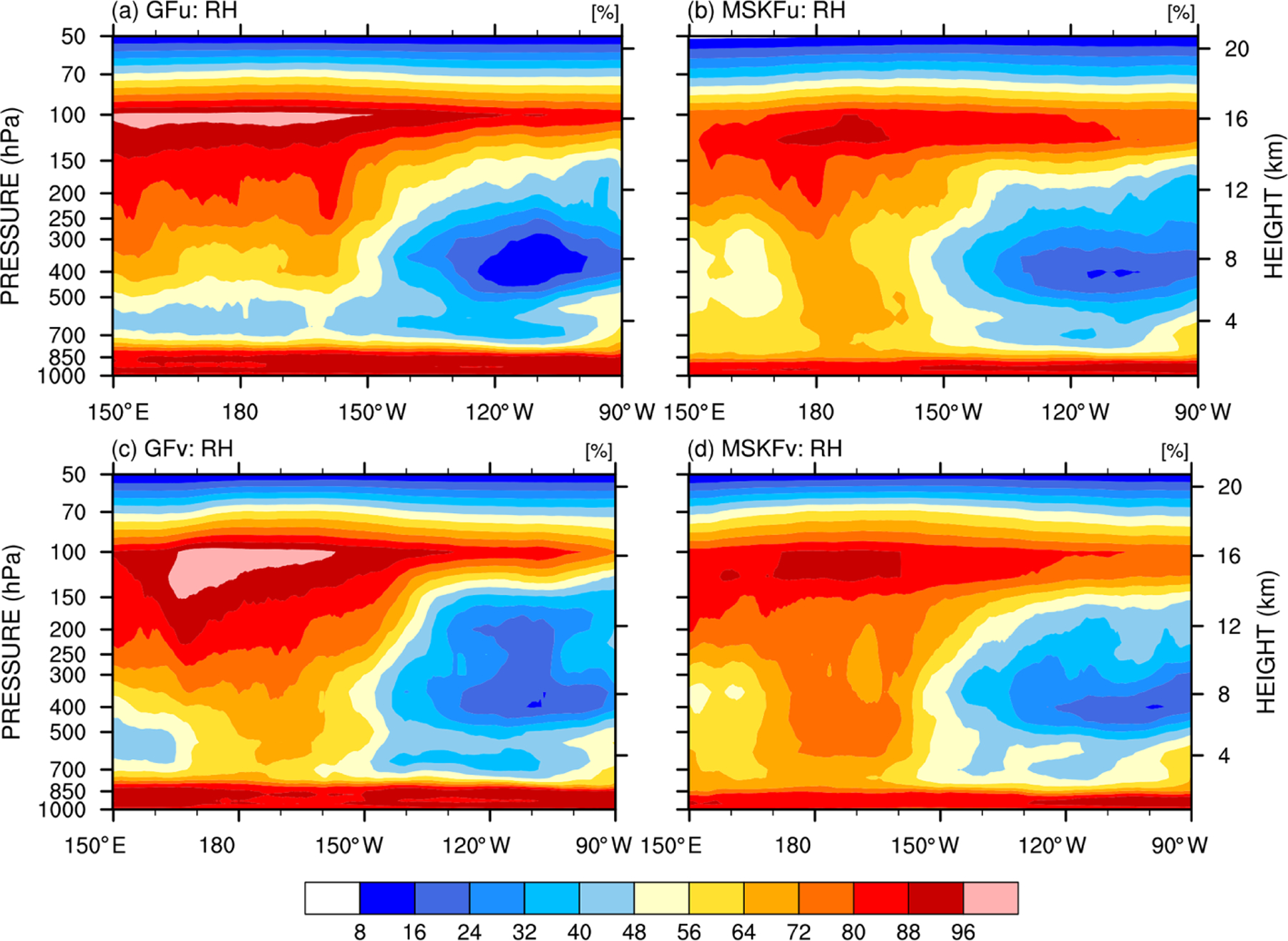
Longitude versus pressure cross section of latitudinally averaged (between 5° S and 5° N) relative humidity (RH) across the tropical Pacific Ocean simulated in (**a, b**) GFu and MSKFu and (**c, d**) GFv and MSKFv for December 2015.

One effect of local mesh refinement is the decreased contribution of parameterized convection compensated by increased contribution of grid-scale cloud microphysics to condensation processes and cloud formation with increasing spatial resolution. Therefore, prior to comparing the simulated LWP and IWP against SSF data, we first investigate differences in relative humidity (RH) between our uniform- and variable-resolution experiments. Figure 11 displays the monthly-mean longitude-pressure cross sections of RH latitudinally averaged between 5° S and 5° N. East of 150° W over the Tropical Eastern Pacific, the four experiments display similar vertical distributions of RH, with relatively lower RH between 700 and 150 hPa and higher RH in the PBL below 700 hPa and in the upper-troposphere above 150 hPa. All four experiments show significant increase in RH west of 150° W across the entire troposphere, over the warm pool where the warmest SSTs are seen (Fig. 2a) and deepest convective updrafts are formed. Comparing GFu against MSKFu over the warm pool shows that GF has stronger drying than MSKF in the lower troposphere, leading to a lower RH between 850 and 300 hPa in GFu than in MSKFu. In addition, GF produces stronger moistening than MSKF in the upper troposphere leading to a higher RH between 300 and 100 hPa in GFu than in MSKFu. As seen in Fig. 11c and d, reducing parameterized deep convection, while enhancing grid-scale cloud microphysics produces a higher RH over the refined area in GFv and MSKFv but without significantly modifying RH over the coarse area of the mesh. Variations in the vertical distribution of RH at pressures less than 400 hPa are more pronounced between GFu and GFv than between MSKFv and MSKFu. Because the cloud fraction (CF) is a function of RH, as described in Xu and Randall (1996; Eq. 1), there is a strong relationship between the longitude-pressure cross sections of RH and CF, as seen in Fig. S2 (see Supplement). The highest CF coincides with the highest RH at about 100 hPa over the warm pool in all four experiments. As for RH, GFu and GFv display higher and lower values of CF than MSKFu and MSKFv in the upper and lower troposphere. The top and bottom panels of Fig. S3 show differences in RH and CF between GFv and GFu and between MSKFv and MSKFu. One notable difference is a stronger increase in upper-tropospheric clouds between MSKFu and MSKFv than between GFv and GFu, particularly over the refined area of the mesh. While increased grid-scale condensation over the refined area impacts the entire tropospheric in GFv, it more strongly affects the upper-troposphere in MSKFv.

**Figure 12.**
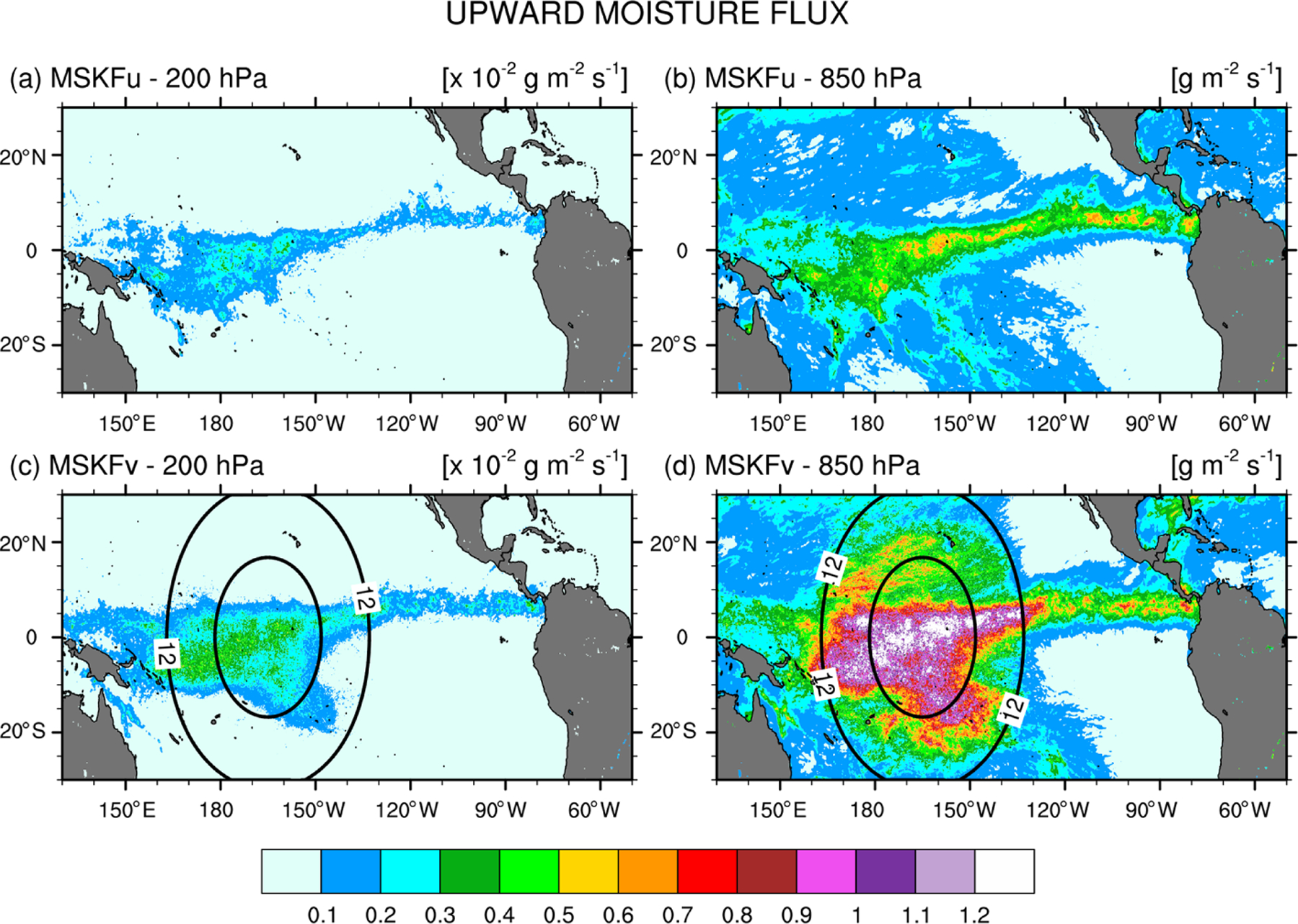
(**a, c**) 200 hPa and (**b, d**) 850 hPa monthly-mean upward moisture flux simulated with MSKF over the tropical Pacific Ocean for December 2015. Top panels (**a**) and (**b**) are for MSKFu, and bottom panels (**c**) and (**d**) are for MSKFv. Note the 1×10^−2^ scaling between 200 and 850 hPa.

To explain the change in RH over the refined area between the uniform- and variable-resolution experiments, we compare the monthly-mean upward moisture flux at 850 and 200 hPa between MSKFu and MSKFv over the Tropical Eastern Pacific (Fig. 12). There is a significant decrease in the upward moisture flux between 850 and 200 hPa in conjunction with decreased specific humidity with height in MSKFu and MSKFv (Fig. 11). As seen in Fig. 12a and b, MSKFu yields highest values of the upward moisture flux along the ITCZ and over the warm pool in association with parameterized deep convection. Outside the ITCZ and warm pool, lower values of the upward moisture flux at 850 hPa result because of reduced deep convection in conjunction with shallow convection, as seen over the SPCZ. At increased spatial resolution, convective processes transition from being parameterized to resolved, producing larger grid-scale vertical velocities, stronger upward moisture flux, and increased grid-scale condensation through the entire troposphere over the refined area of the mesh. Comparing Fig. 12c and d versus Fig. 12a and b outlines the intensification of vertical moisture transport at both pressure levels over the refined area, leading to the increased relative humidity with increased spatial resolutions shown in Fig. 11.

### 5.2 Liquid water path (LWP)

**Figure 13.**
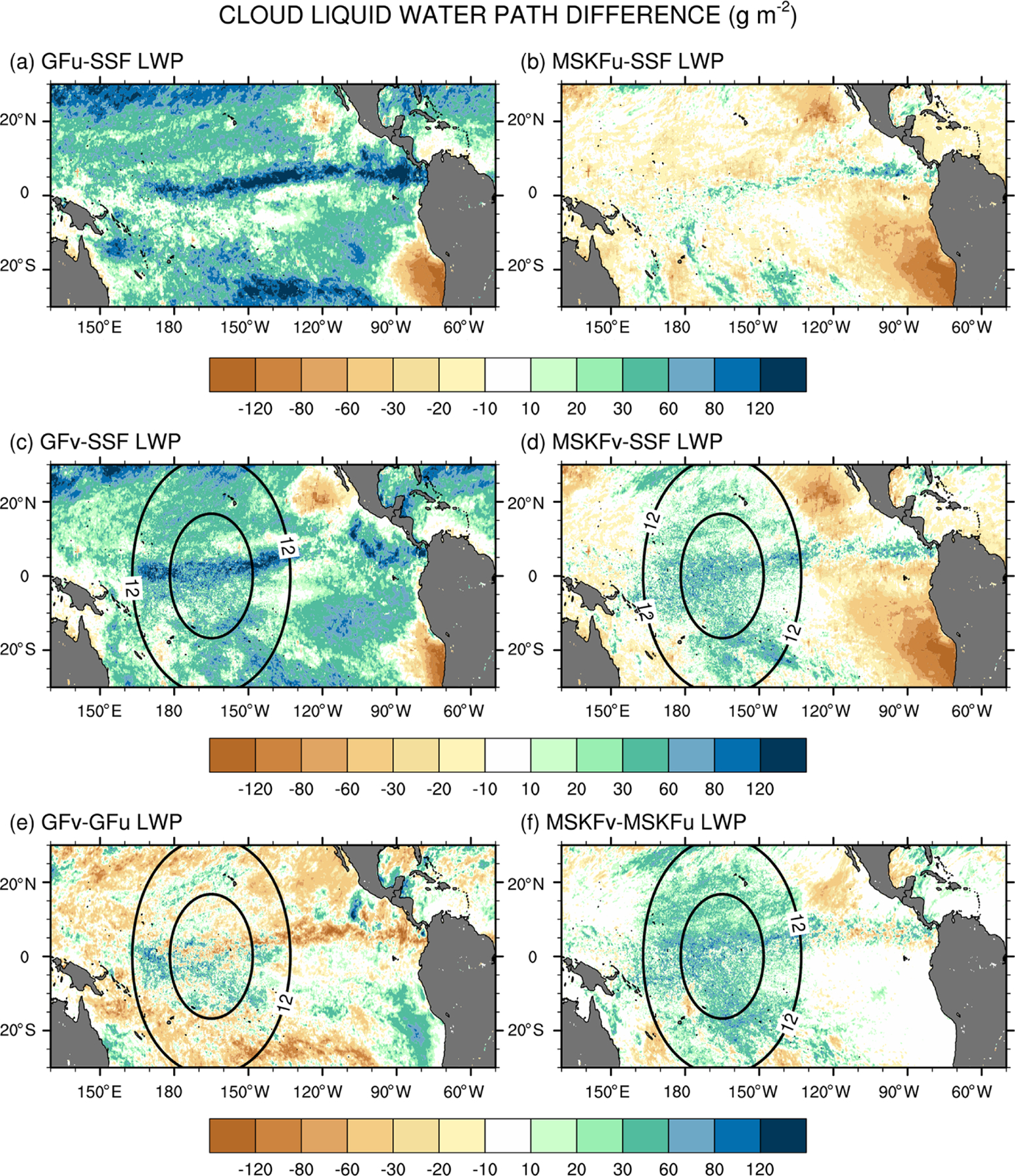
Monthly-mean cloud liquid water path (LWP) difference over the tropical Pacific Ocean between (**a, b**) GFu (MSKFu) and SSF data and (**c, d**) GFv (MSKFv) and SSF data and monthly-mean LWP difference between (**e, f**) GFv (MSKFv) and GFu (MSKFu) for December 2015.

Figure 13 displays difference maps between the simulated and satellite-derived LWP and between GFv (MSKFv) and GFu (MSKFu). In Fig. 13, the simulated LWP is calculated using only the grid-scale cloud liquid water mixing ratio from THOM. Separate analyses would show that adding the prognostic grid-scale rain mixing ratio to the simulated LWP further increases biases when compared against the SSF LWP (not shown for brevity). We also do not include the contribution of the convective cloud liquid water mixing ratio to the LWP, which is small compared to that from the grid-scale cloud microphysics. Figure 13 highlights that GFu strongly overestimates the LWP over the ITCZ and between 20° N (20° S) and the northern (southern) limits of our analysis. As seen in Fig. 6, GFu attempts to form low-level boundary layer clouds off the coast of Peru, but these clouds form too far west from the coast when compared against observations. This same bias is depicted in Fig. 13a since these low-level boundary layer clouds are characterized by high LWP. In Fig. 13b, decreased bias between the MSKFu and SSF LWP reflects that the LWP is strongly decreased in MSKFu compared to GFu, outside of the areas of low-level boundary layer clouds. If we set aside that MSKFu is unable to simulate low-level clouds off the Baja California Peninsula and coast of Peru, the magnitude and regional patterns of the LWP simulated in MSKFu is in fairly good agreement with the SSF LWP. Because MSKF does not allow deep and shallow convection to coexist within the same grid cell, and deep convection dominates shallow convection over the ITCZ and warm pool, we suggest that detrained cloud water from deep convection as a source for grid-scale microphysics contributes a major part to the LWP produced by MSKFu. Figure 13e and f reveal that the mesh refinement impacts the LWP simulated with MSKF more effectively than that simulated with GF inside the refined area. This result is in agreement with the stronger increase in RH between MSKFu and MSKFv than between GFu and GFv at lower levels. MSKFv yields an increased LWP relative to MSKFu over the entire refined area (Fig. 13f). MSKFv also has increased LWP compared to MSKFu over the coarse area but not as large as that seen over the refined area. Figure 13e shows that the LWP differences do not have a strong positive or negative trend inside the refined area, due to the fact that GF allows deep and shallow convection to coexist within the same grid cell of deepest convective activity, mainly over the ITCZ and warm pool, and shallow convection does not account for variations in horizontal grid spacing. Over the coarse area, an obvious decrease in the LWP between GFv and GFu is seen over the ITCZ in the Tropical Eastern Pacific as well as along the southern boundary of our analysis.

In order to investigate the reasons why the LWP simulated in GFu strongly exceeds that from the SSF product and MSKFu, we calculate the monthly-mean LWP produced in grid cells with incidence of deep convection, shallow convection, and no convection, using LWP hourly outputs from GFu. Separate maps show that a major fraction of the LWP over convectively active regions such as the ITCZ is actually produced at times when no convection is active or when only shallow convection is triggered (not shown for brevity). In GF, and in contrast to deep convection, shallow convection detrains total water as a source of grid-scale water vapor instead of detraining water vapor, cloud liquid, and ice water separately. Because the detrained total water is treated as a source of water vapor, supersaturation conditions are more likely to persist and later removed by grid-scale condensation. In contrast, detrainment from deep convective updrafts acts as a source of liquid water if temperatures are warmer than 258K. Deep convection in conjunction with grid-scale condensation contributes the least to the LWP because updrafts are taller and their cloud-top temperatures colder than those from shallow convection, leading to condensation and deposition to occur at levels where temperatures are colder than 258K and where ice-phase processes dominate.

**Figure 14.**
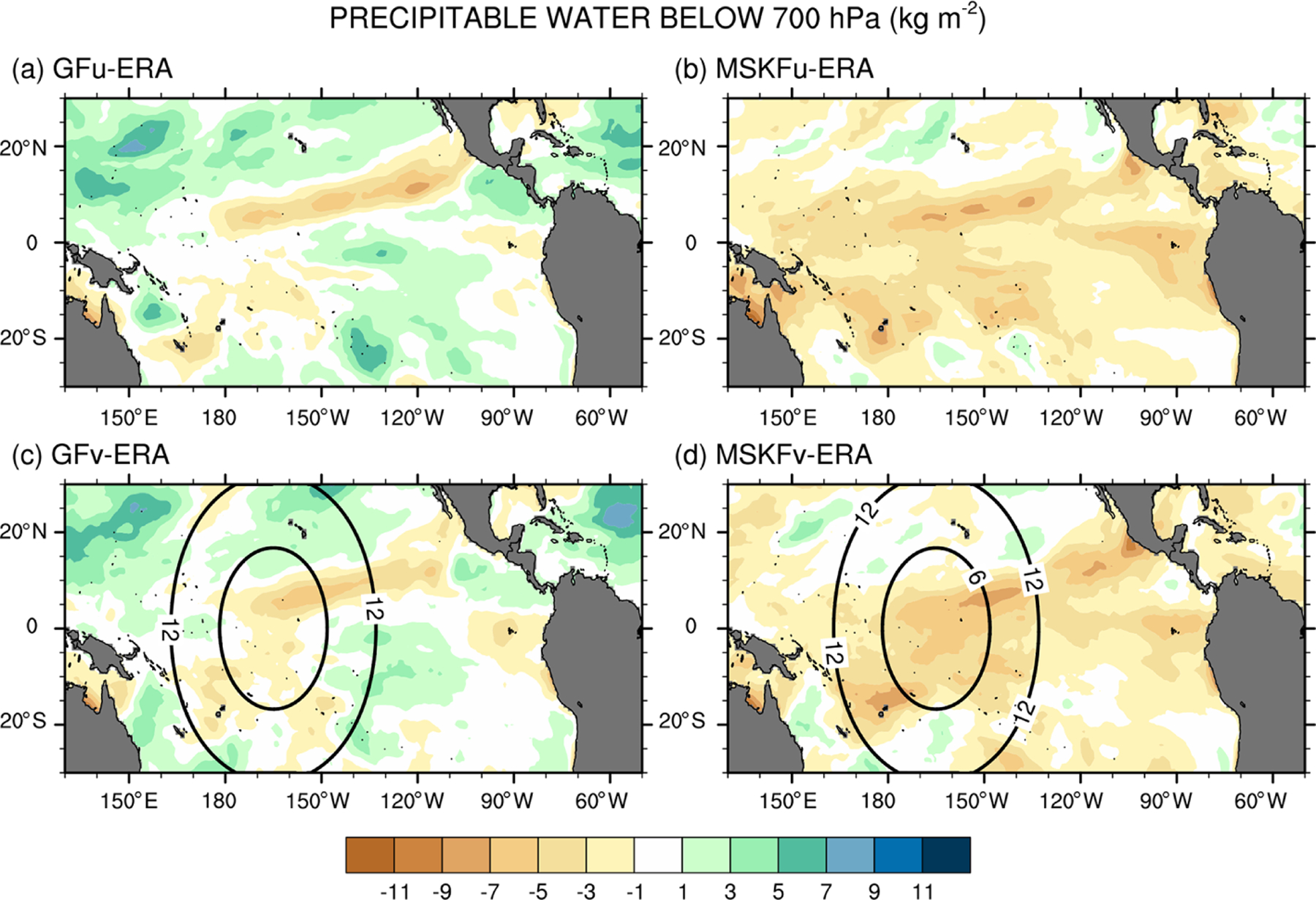
Monthly-mean difference between the simulated and ERA-Interim precipitable water below 700 hPa over the tropical Pacific Ocean for December 2015.

The impact of more active shallow convection in GFu (GFv) than in MSKFu (MSKFv) is analyzed using Fig. 14, which shows differences in the monthly-mean precipitable water below 700 hPa between our experiments and ERA-Interim reanalyses. Because varying horizontal resolution does not affect shallow convection, GFv (MSKFv) displays similar biases as GFu (MSKFu) over the entire analysis domain, including the refined area. Comparing Fig. 14a and c versus Fig. 14b and d reveals that the precipitable water simulated in GFu (GFv) displays a positive bias, whereas that simulated in MSKFu (MSKFv) displays a negative bias in the lower troposphere relative to ERA-Interim data, mainly over areas of shallow convection. In GF, the abundance of shallow convection (Fig. 6a, c) associated with detrained total water acting as a source of grid-scale water vapor promotes the lower troposphere to stay more humid and cloud liquid water to form more often than actually observed (Fig. 13a, c), north and south of the ITCZ and warm pool. In MSKF, while shallow convection is as widespread over the tropical Pacific Ocean as in GF, it cannot act as a major source of detrained total water to the grid-scale microphysics because it is not triggered as often as deep convection. In addition, because MSKF partitions detrained water into water vapor, cloud water, cloud ice, rain, and snow, instead of detraining total water in the form of water vapor as in GF, the amounts of available water vapor and cloud liquid water are reduced relative to GF.

### 5.3 Ice water path (IWP)

**Figure 15.**
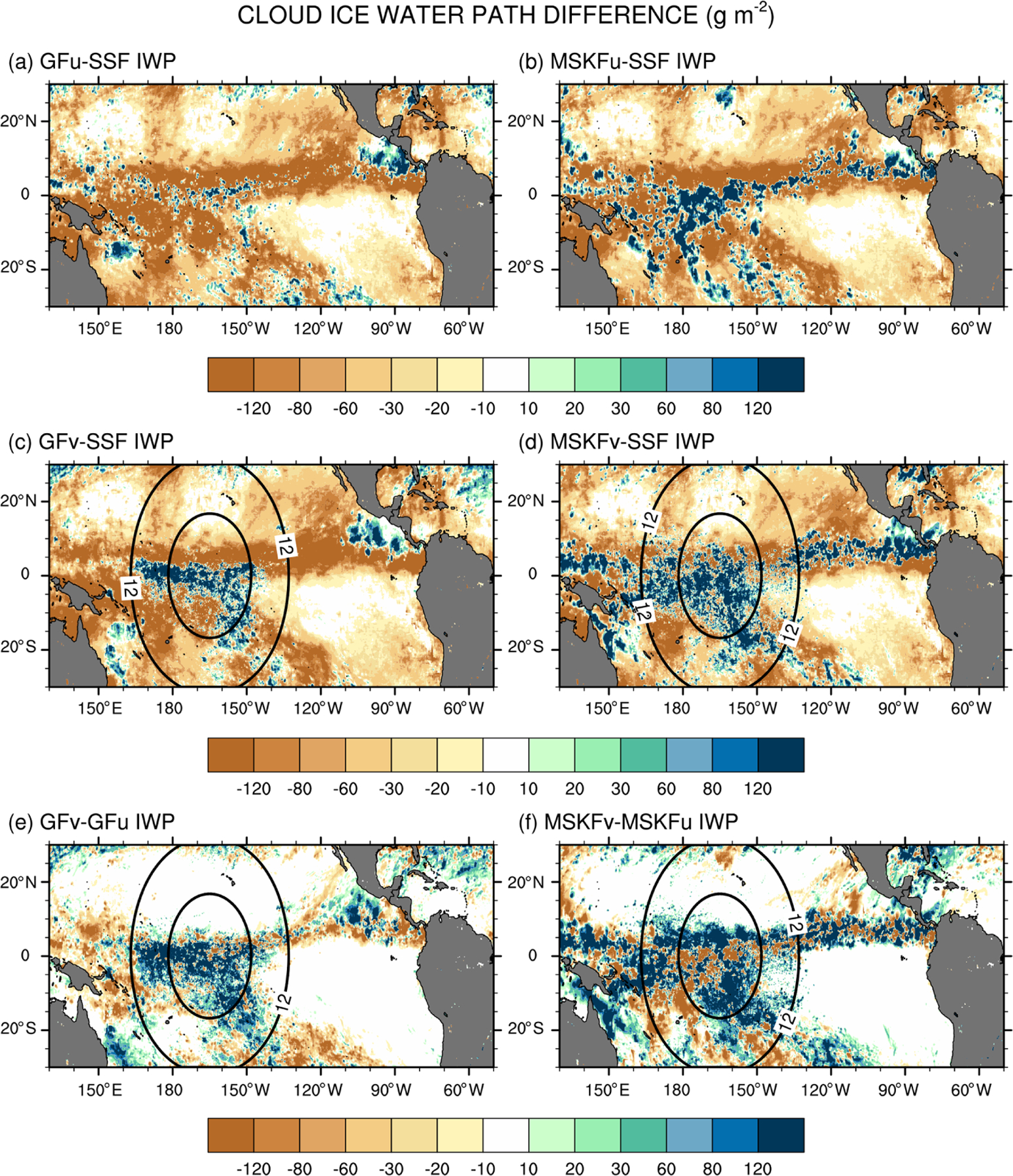
As Fig. 13 but for the cloud ice water path (IWP).

Because MODIS is relatively insensitive to precipitation, the simulated IWP should comprise cloud ice, snow, and graupel. Because graupel contributes a minor part to the IWP relative to cloud ice and snow, and our results highlight strong biases against SSF data, we do not include graupel in our computation of the simulated IWP. It is also important to note that because THOM has the propensity to rapidly convert cloud ice to snow (Thompson et al., 2016), most of the IWP is in the form of snow, which falls at higher speeds than cloud ice, enhancing the depth of ice clouds. Lastly, Fig. 5c and d show that our gridding of the IWP orbital data produces increased monthly-mean IWP compared to the official SSF1deg product. This result implies that biases between the simulated and satellite-derived IWP will be underestimated when using our SSF 0.2°×0.2° IWP data. Figure 15 shows difference maps between the simulated and satellite-derived IWP and between GFv (MSKFv) and GFu (MSKFu). When compared against the SSF IWP, GFu is the only experiment that mostly underestimates the IWP along the ITCZ and warm pool, whereas GFv yields a strong increase in the IWP over the refined area of the mesh relative to GFu. Both GFu and GFv overestimate the IWP along the west coast of Central America, as they did for the LWP and precipitation. Comparing MSKFu (MSKFv) against GFu (GFv) shows that MSKF leads to increased positive biases in the IWP compared to GF over the entire ITCZ and warm pool. Increased convective detrainment of cloud ice as a source of grid-scale cloud ice to THOM in MSKF compared to in GF, because partitioning between cloud liquid and ice water starts at warmer temperatures, may be responsible to the increased IWP. Figure 15e and f reveal that increasing spatial resolution worsens the simulated IWP compared to the SSF IWP over the refined area in GFv and MSKFv. As shown in Fig. 11, mesh refinement over the warm pool yields higher upper-tropospheric relative humidity leading to increased ice cloud microphysics. In contrast to GFv, MSKFv displays an increase in the IWP over the coarse area of the mesh, showing a stronger impact of the refined area on the coarse area of the mesh in MSKFv than in GFv in the upper-troposphere.

### 5.4 TOA radiation budget

Biases in the LWP and IWP introduce biases in the cloud fraction and cloud optical properties, which in turn lead to biases in the simulated TOALW and TOASW compared to CERES-SSF data. Figures S4, S5, and S6 display the monthly-mean CF, TOALW, and TOASW from SSF data for December 2015 and the differences between the model results and observations. Focusing on areas of deep convection over the ITCZ and warm pool, all four simulations overestimate CF, with larger biases seen in the GF than the MSKF experiments and larger biases seen in the variable-resolution than the uniform-resolution experiments. All four simulations also overpredict CF along the west coast of Central America, while underpredicting CF over areas of stratiform clouds along the west coast of South America and the Baja California Peninsula. The impact of CF biases is that all four experiments underestimate the size of the warm pool and width of the ITCZ, leading the TOALW (TOASW) to be too high (low) over areas of deep convection. These differences are clearly linked to the differences noted in the LWP and IWP between MPAS and SSF data.

## 6 Discussion

When running GFu (MSKFu) and GFv (MSKFv), we set the time step to be as large as possible to reduce the computational cost of the various experiments without compromising computational stability. Using decreased time steps between the uniform- and variable-resolution experiments from 150 to 30 s implies that it is not possible to directly compare the mean state of GFv (MSKFv) against that of GFu (MSKFu) in the coarse area of the variable-resolution mesh and analyze upscaling effects of local mesh refinement. This is in contrast to Sakaguchi et al. (2015) and Hagos et al. (2013), which constrain the time step to be the same at all horizontal scales, allowing the study to assess the upscaling effect of mesh refinement across the transition zones between the refined and coarse areas of the mesh and far from the refined mesh. In order to understand the increase in convective precipitation east and west of the transition zones in GFv relative to GFu, we run GFu with the reduced 30 s time step to quantify the dependence of convective precipitation to the dynamic time step. As seen in Fig. S7a (S7b), reducing the time step from 150 to 30 s strongly increases convective precipitation over convectively active regions of the tropical Pacific Ocean, highlighting the sensitivity of GF to the time step. Reducing the time step in MSKFu yields convective precipitation differences that are not as large as those seen in Fig. S7b (not shown for brevity). Using the Community Atmosphere Model Version 4 (CAM4) with a T340 spectral truncation and a 5 min time step, Williamson (2013) demonstrates the dependence of the removal of supersaturation conditions to the shallow (30 min) and deep (1h) convective timescales. While it is important to point out that the sensitivity studies discussed in Williamson (2013) depend on the CAM4 coupling between the convective and grid-scale cloud parameterizations and the dynamical core, shorter convective timescales relative to the time step yield faster removal of moist instabilities through vertical motions and condensation. In GF, the timescales used in the *AS* and *KF* closures are set to the dynamical time step and 20 min, respectively. While the contribution of the *KF* closure decreases by a factor of 5 in response to the decreased time step, the contribution of the *AS* closure is independent of the convective timescale but will affect the cloud-base mass flux through variations in the cloud work function. In order to further understand the impact of the time step on increased supersaturation and convective precipitation in GF, a detailed analysis of the contributions of the dynamics and physics forcing on the *AS* cloud work function in MPAS is needed. This is the object of future research.

## 7 Summary and future research

Uniform- and variable-resolution experiments with two scale-aware parameterizations of deep convection (GF and MSKF) in MPAS yield significant biases between the simulated and satellite-derived monthly-mean precipitation rates, LWP, IWP, and CF over the tropical Pacific Ocean for December 2015. In turn, biases affect the cloud fraction and optical properties producing significant differences in the TOALW and TOASW compared to CERES-SSF data.

Tropical precipitation simulated with uniform-resolution experiments is overestimated compared to TMPA, due to subgrid-scale deep convection. Biases using GF are as large as those using MSKF and result in part because the simulated ITCZ is located south of its observed location. Variable-resolution experiments do not produce significant improvement in simulating precipitation against TMPA. Inside the refined area, decreased convective precipitation plus compensating increased grid-scale precipitation have the simulated total precipitation to exhibit similar biases between the uniform- and variable-resolution experiments with GF and MSKF. One major difference in using GF instead of MSKF is the strong upscaling effect of the refined mesh on the coarse mesh, producing a strong increase in convective precipitation east and west of the refined mesh. Because deep convection does not exhibit similar behavior over the transition zone between the coarse and refined areas of the mesh in MSKF, we plan further to investigate this difference in convective precipitation in terms of the size of convective updrafts as a function of horizontal resolution and increased moistening of the lower troposphere from shallow convection.

Differences in the simulated LWP between the uniform- and variable-resolution experiments using GF and MSKF against the CERES-SSF LWP highlight the need to revise the treatment of shallow convection to improve warm-phase clouds in both schemes. While experiments using MSKF yield the simulated LWP to be in reasonable agreement against that from the CERES-SSF product, those using GF yield the simulated LWP to be strongly overestimated. Analyses show that shallow convection and cloud microphysics processes explain most of the increased LWP in GFu and GFv compared to MSKFu and MSKFv and satellite-derived data. We plan to update the GF shallow convection scheme with that implemented in version 4.1 of the Advanced Research WRF (Skamarock et al., 2008) model. Because the updated scheme includes an improved cloud model that allows water vapor and cloud liquid water to detrain separately and a fraction of condensed water to precipitate, we will focus on the impact of explicit detrainment of cloud liquid water and precipitation from shallow convective updrafts on the simulated LWP in GF. Results show that MSKF underestimates shallow convection, leading the troposphere below 700 hPa to be drier than actually observed. These results imply that the shallow convection scheme in MSKF needs to be updated or that a separate parameterization of shallow convection needs to be used in addition to that in MSKF. Using the same parameterization of shallow convection and partitioning of the detrained condensed water between cloud liquid water and ice in GF and MSKF will further provide understanding in the partitioning of the LWP between subgrid-scale deep and shallow convection. Variable-resolution experiments strongly overestimate the IWP compared to CERES-SSF data over the refined area of the mesh, leading to strong biases in the cloud fraction and TOA longwave and shortwave radiation. Because subgrid-scale deep convection is reduced at increased horizontal resolutions, grid-scale cloud microphysics contributes a major part to biases in the simulated IWP.

Parameterizing the dependence of subgrid-scale deep convection as a function of horizontal resolution allows the use of variable-resolution meshes spanning between hydrostatic and nonhydrostatic scales within a global framework for regional NWP and climate experiments. Although deep convection is not fully explicitly resolved over the refined area of the mesh in our variable-resolution experiments, it is substantially reduced relative to that over the coarse area of the mesh, allowing the contribution of subgrid-scale convection and cloud microphysics processes to be contrasted. As horizontal resolution increases from the coarse to refined area of the mesh, deep convection gradually transitions from parameterized to resolved, and cloud microphysics contribute a major part to moist processes over the refined mesh. Shallow convection coupled with grid-scale microphysics contributes a major part to the low-level cloud liquid water and mixed-phase clouds, whereas grid-scale cloud microphysics contribute a major part to the formation of upper-tropospheric ice clouds over the refined area. Our results show that mesh refinement does not systematically improve precipitation and clouds over the tropical Pacific Ocean as grid-scale condensation increases at increased resolutions. As cloud microphysics processes drive the moisture budget over the refined area of the mesh, we propose expanding this diagnostic study to a process study by further understanding the cloud microphysics processes that need to be improved in order to reduce discrepancies between model and observations. In that vein, the recently developed MSKF that includes a double-moment microphysics (Glotfelty et al., 2019) would be useful in a future process study.

## Supplementary Material

Sup1

## Figures and Tables

**Table 1.** Horizontal mesh resolution, minimum and maximum distance between grid-cell centers, time step, horizontal diffusion length scale, and convective parameterization (CP) for numerical experiments with the uniform- and variable-resolution meshes.

	GFu	MSKFu	GFv	MSKFv
No. of cells	655 362	655 362	1 622 018	1 622 018
Min. cell distance (km)	22.8	22.8	4.4	4.4
Max. cell distance (km)	31.8	31.8	37.8	37.8
Time step (s)	150	150	30	30
Minimum diffusion	30	30	6	6
length scale (km) CP	GF	MSKF	GF	MSKF

**Table 2.** Area-averaged incidence of deep and shallow convection. The REFINED and EAST areas are shown in Fig. 9a.

	Deep convection (%)		Shallow convection (%)	
	REFINED	EAST	REFINED	EAST
GFu	20	30	52	52
GFv	23	36	47	48
MSKFu	27	33	14	17
MSKFv	10	36	17	15

**Table 3.** Area-averaged convective, grid-scale, and total precipitation rates over the same areas as those described for Table 2. The REFINED and EAST areas are shown in Fig. 9a.

	Convective (mm d^−1^)		Grid-scale (mm d^−1^)		Total (mm d^−1^)	
	REFINED	EAST	REFINED	EAST	REFINED	EAST
GFu	10.0	8.7	6.1	3.7	16.1	12.4
GFv	1.9	14.0	12.1	1.1	14.0	15.1
MSKFu	10.9	10.6	4.9	4.8	15.8	15.5
MSKFv	1.7	11.1	11.8	5.4	13.5	16.5
TMPA					20.7	7.3
