# Supplementary material for: Impact of scale-aware deep convection on the cloud liquid and ice water paths and precipitation using the Model for Prediction Across Scales (MPAS-v5.2)
Source: Geosci Model Dev. Author manuscript; Available in PMC 2021 Jun 29. (PMC7970523; doi:10.5194/gmd-13-2851-2020)
Supplement: Sup1 [file NIHMS1612072-supplement-Sup1.pdf]

Supplement of Geosci. Model Dev., 13, 2851–2877, 2020
https://doi.org/10.5194/gmd-13-2851-2020-supplement
© Author(s) 2020. This work is distributed under
the Creative Commons Attribution 4.0 License.

Geoscientific Model Development
Open Access

Supplement of

Impact of scale-aware deep convection on the cloud liquid and ice water paths and precipitation using the Model for Prediction Across Scales (MPAS-v5.2)

Laura D. Fowler et al.

Correspondence to: Laura D. Fowler (laura@ucar.edu)

The copyright of individual parts of the supplement might differ from the CC BY 4.0 License.

List of Figure Captions for Supplemental Figures

Figure S1: Monthly-mean cloud liquid water path (LWP, top panels), cloud ice path (IWP, middle panels), and cloud fraction (CF, bottom panels) over the Tropical Pacific for December 2015 from the Terra satellite. Panels (a), (c), and (e) are for the lower cloud layer; panels (b), (d), and (f) are for the upper cloud layer.

Figure S2: Longitude versus pressure cross-section of monthly-mean latitudinally-averaged cloud fraction (CF) across the Tropical Pacific Ocean simulated in GFu and MSKFu (top panels) and GFv and MSKFv (bottom panels) for December 2015.

Figure S3: Longitude versus pressure cross-section difference in the monthly-mean relative humidity (RH; top panels) and monthly-mean cloud fraction (CF; bottom panels) over the Tropical Pacific Ocean between GFv and GFu, and between MSKFv and MSKFu for December 2015.

Figure S4: Monthly-mean vertically-integrated cloud fraction (TOACF) over the Tropical Ocean from a) CERES-SSF data, and difference in the TOACF between GFu (MSKFu) and CERES-SSF (middle panels) and between GFv (MSKFv) and CERES-SSF (bottom panels) for December 2015.

Figure S5: Monthly-mean TOA upward longwave radiation (TOALW) over the Tropical Ocean from a) CERES-SSF data, and difference in the TOALW between GFu (MSKFu) and CERES-SSF (middle panels) and between GFv (MSKFv) and CERES-SSF (bottom panels) for December 2015.

Figure S6: As Fig. S5, but for the TOA net shortwave radiation (TOASW)

Figure S7: 10-day mean convective precipitation rate over the Tropical Pacific Ocean (a) simulated with GFu using a 30s time-step; (b) difference between GFu using a 30s and 150s timestep; and (c) ratio between GFu using a 30s time-step and GFv.

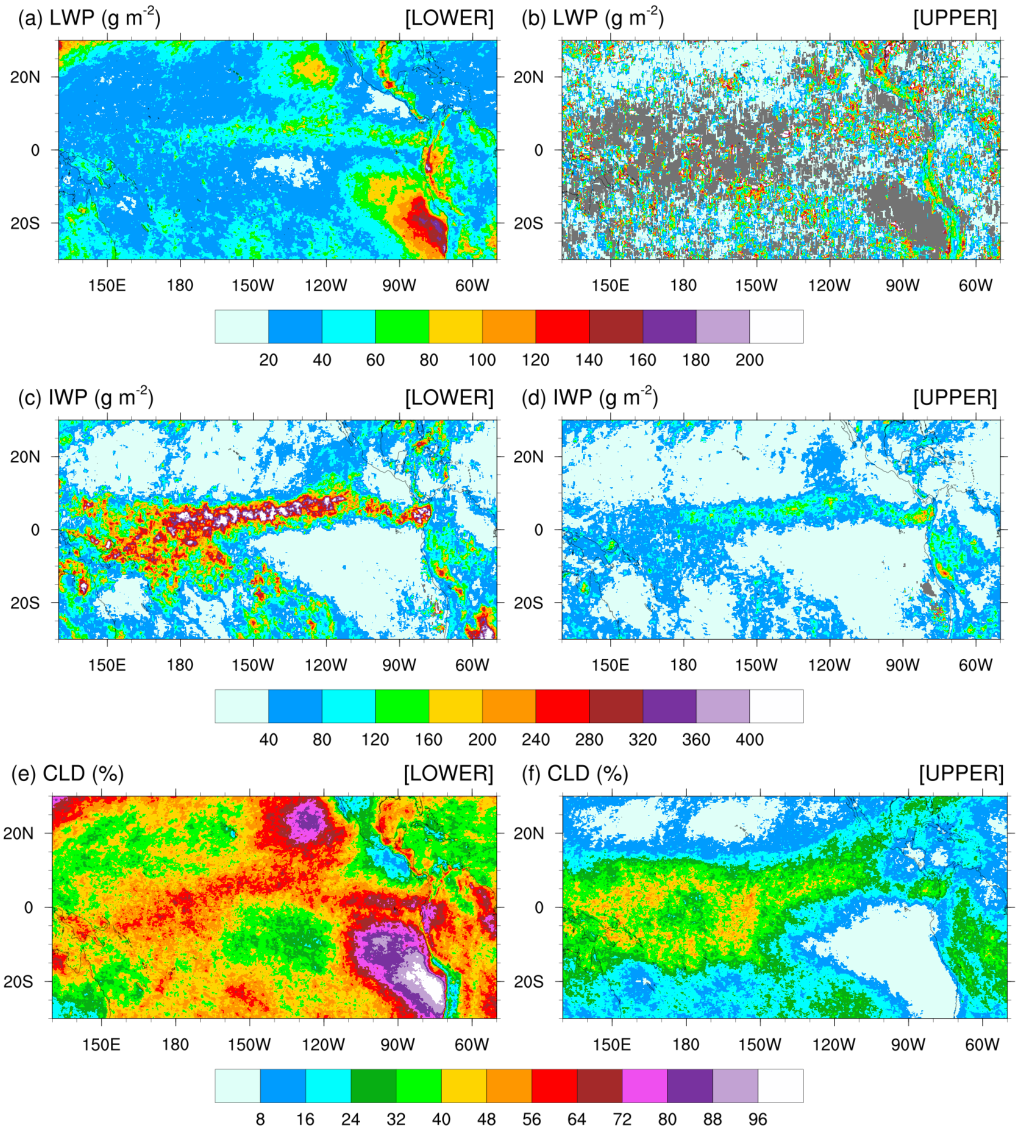

Figure S1: Monthly-mean cloud liquid water path (LWP, top panels), cloud ice path (IWP, middle panels), and cloud fraction (CF, bottom panels) over the Tropical Pacific for December 2015 from the Terra satellite. Panels a), c), and e) are for the lower cloud layer; panels b), d), and f) are for the upper cloud layer.

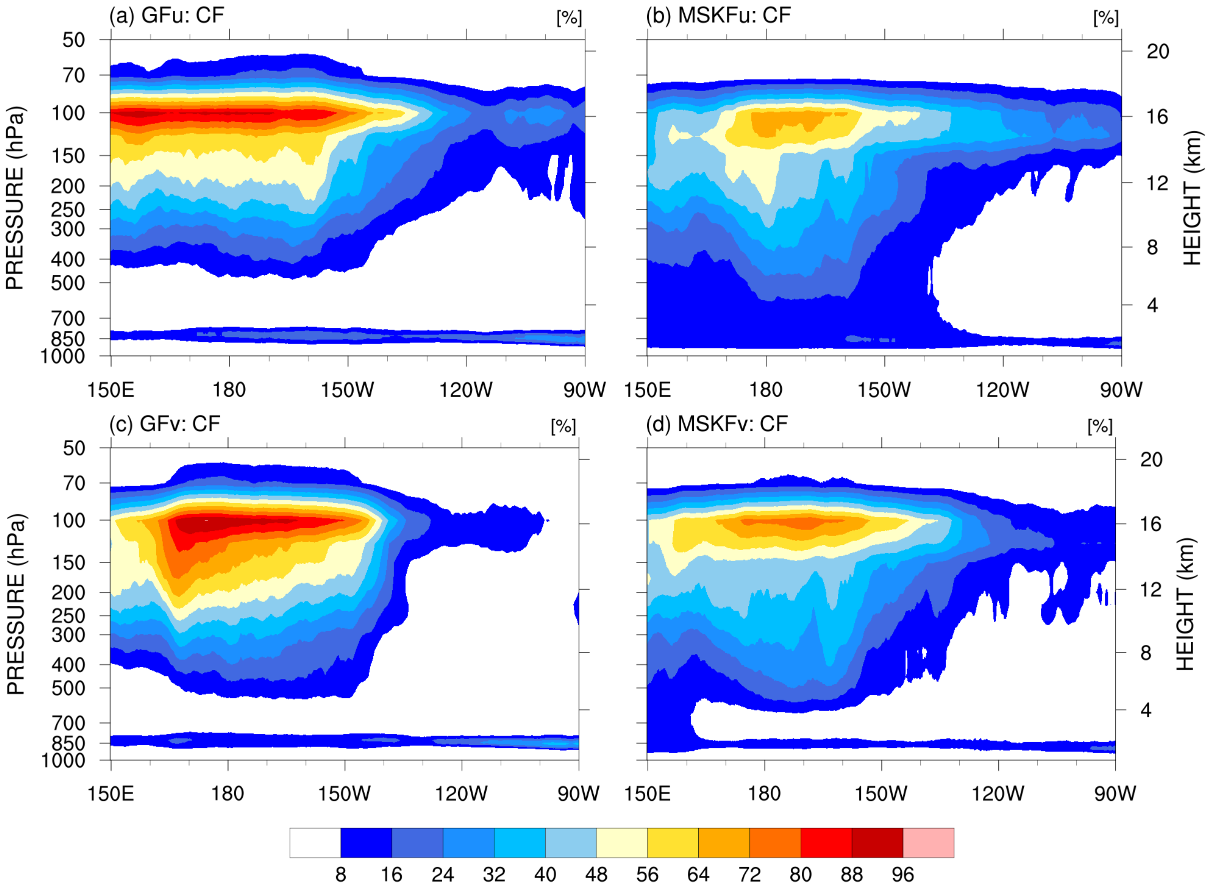

Figure S2: Longitude versus pressure cross-section of monthly-mean latitudinally-averaged cloud fraction (CF) across the Tropical Pacific Ocean simulated in GFu and MSKFu (top panels) and GFv and MSKFv (bottom panels) for December 2015.

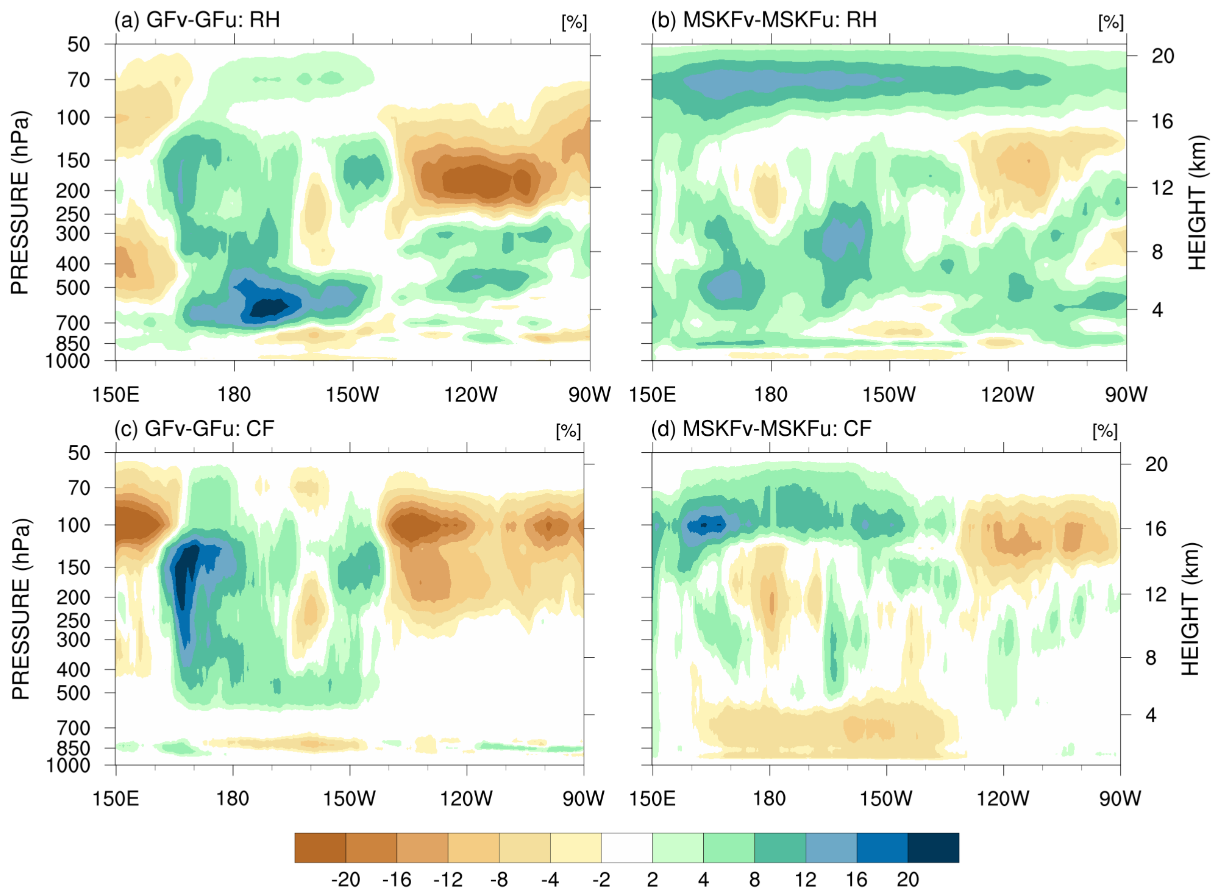

Figure S3: Longitude versus pressure cross-section difference in the monthly-mean relative humidity (RH; top panels) and monthly-mean cloud fraction (CF; bottom panels) over the Tropical Pacific Ocean between GFv and GFu, and between MSKFv and MSKFu for December 2015.

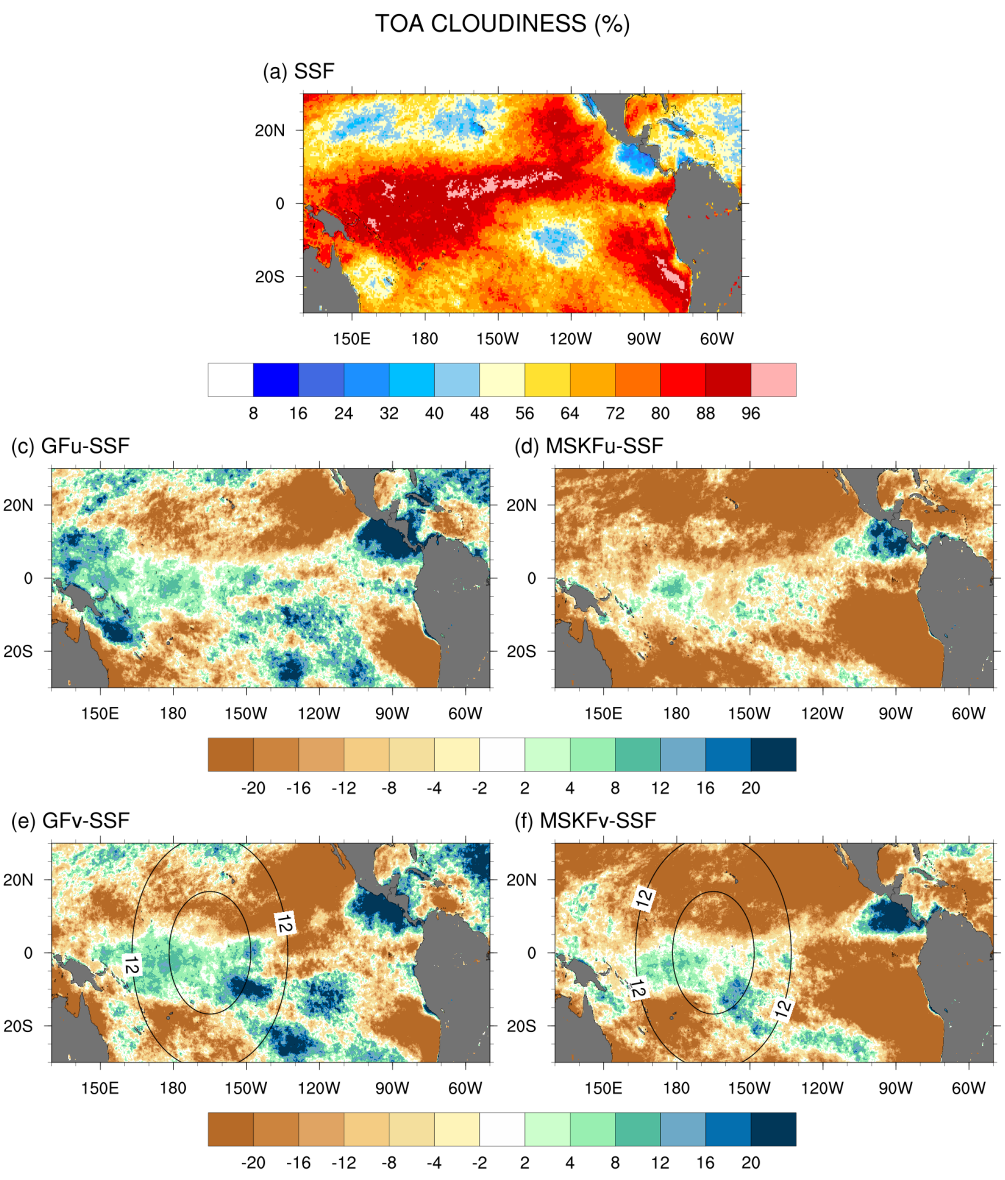

Figure S4: Monthly-mean vertically-integrated cloud fraction (TOACF) over the Tropical Ocean from a) CERES-SSF data, and difference in the TOACF between GFu (MSKFu) and CERES-SSF (middle panels) and between GFv (MSKFv) and CERES-SSF (bottom panels) for December 2015.

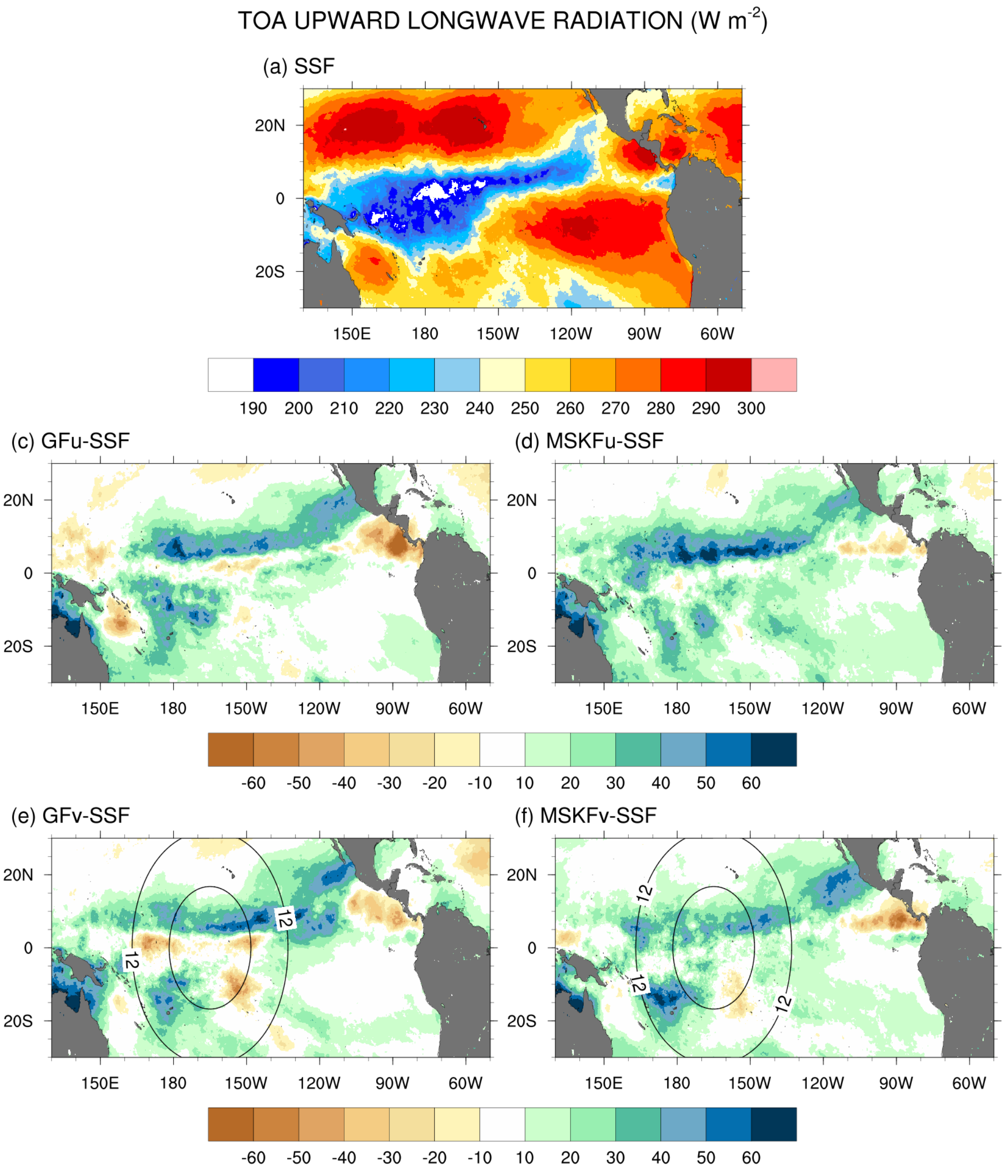

Figure S5: Monthly-mean TOA upward longwave radiation (TOALW) over the Tropical Ocean from a) CERES-SSF data, and difference in the TOALW between GFu (MSKFu) and CERES-SSF (middle panels) and between GFv (MSKFv) and CERES-SSF (bottom panels) for December 2015.

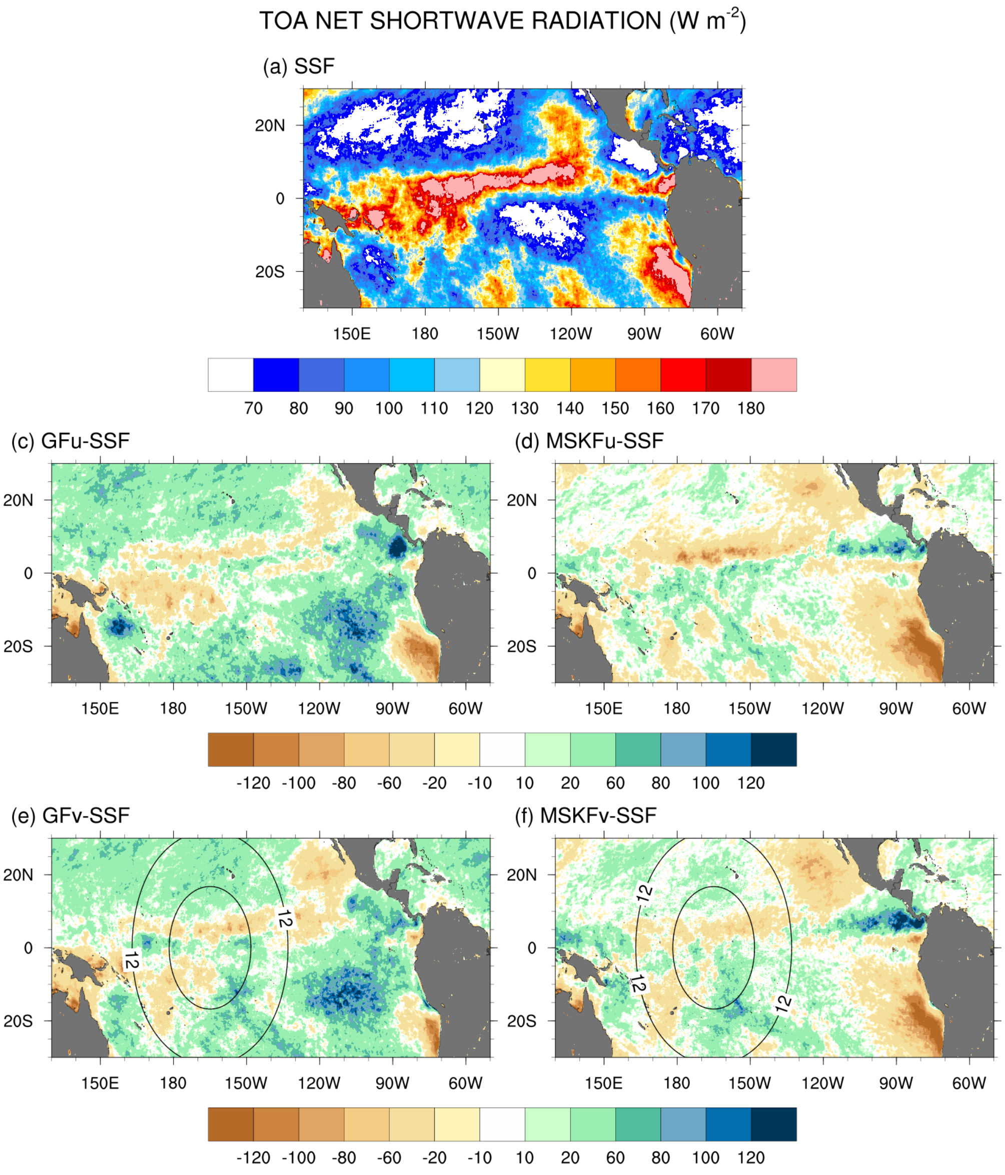

Figure S6: As Fig. S5, but for the TOA net shortwave radiation (TOASW).

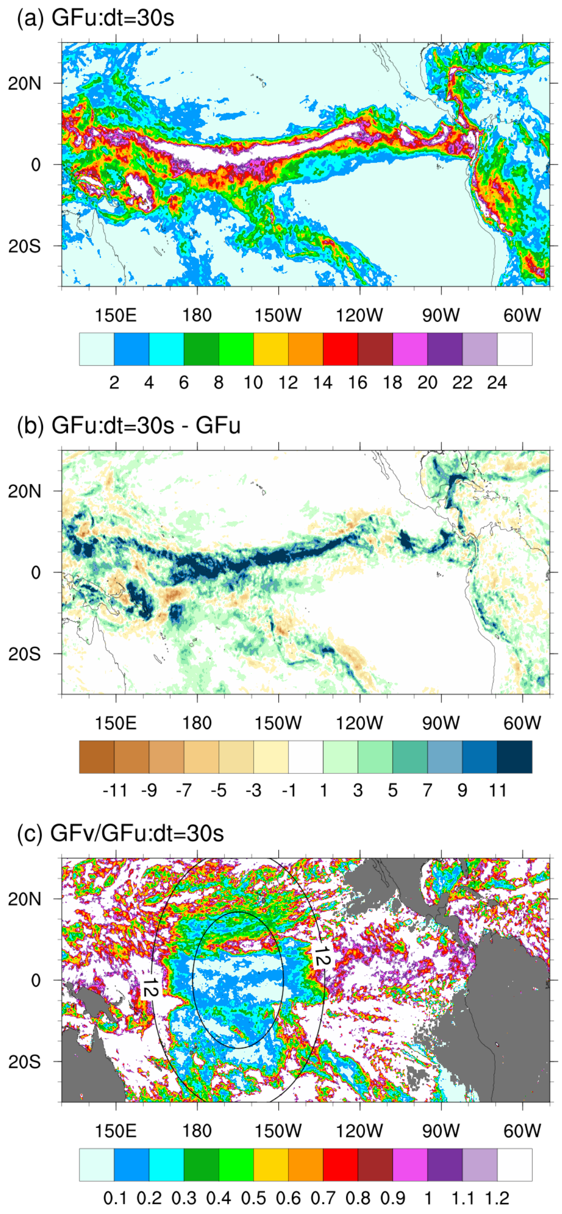

Figure S7: 10-day mean convective precipitation rate over the Tropical Pacific Ocean a) simulated with GFu using a 30s time-step; b) difference between GFu using a 30s and 150s timestep; and c) ratio between GFu using a 30s time-step and GFv.
